# Integrating temperature-dependent tissue properties into focused ultrasound computational models for enhanced treatment planning

**DOI:** 10.1080/02656736.2025.2606701

**Published:** 2026-01-04

**Authors:** Christian Valencia Narva, Allison Payne, Christopher R. Dillon

**Affiliations:** a Department of Mechanical Engineering, Brigham Young University, Provo, UT, USA; b Department of Radiology and Imaging Sciences, University of Utah, Salt Lake City, UT, USA

**Keywords:** Focused ultrasound (FUS), temperature-dependent properties, acoustic properties, computational modeling, necrosis prediction

## Abstract

**Objective:**

Focused ultrasound (FUS) is a noninvasive therapy that can ablate tissues with precision. Computational simulations using the Pennes Bioheat Transfer Equation (PBTE) can aid FUS treatments by predicting temperature distributions. However, traditional models assume constant thermal and acoustic properties, potentially oversimplifying tissue behavior during treatments.

**Methods:**

This study integrates temperature-dependent thermal properties (thermal conductivity, specific heat capacity, and perfusion) into finite-difference time-domain FUS simulations. Three scenarios were simulated: (1) homogeneous liver with both high- and low-power sonications, (2) rabbit thigh muscle validated against preclinical magnetic resonance temperature imaging (MRTI), and (3) an extended simulation of the rabbit thigh with temperature-dependent acoustic properties. Comparisons were made against models using constant-properties collected at 25°C, 20°C, and/or 37°C.

**Results:**

For high-power liver sonications, temperature-dependent properties increased necrosis (tissue volume exceeding 240 CEM43) by 17.6% and 13% compared to constant-property models at 25°C and 37°C, respectively. Low-power sonications had 17–20% lower temperature rises with temperature-dependent properties. In rabbit muscle, temperature-dependent models showed up to 18% difference in necrosis volume, with temperature curves following the trends observed in MRTI. Incorporating temperature-dependent acoustic properties increased the predicted necrosis volume by up to 30%. Updating thermal properties every 2.5 s maintained accuracy (1% difference in peak temperature) while reducing computational cost by 70%.

**Conclusion:**

For FUS simulations involving perfusion shutdown in highly perfused tissues (high-power ablations) or involving hyperemia (low-power hyperthermia), incorporating temperature-dependent properties substantially impacts temperature profiles and necrosis predictions. Properties need not be updated every time step to balance simulation accuracy and computational efficiency.

## Introduction

Focused ultrasound (FUS) is a noninvasive surgical technique [1,2] used to treat diseased tissues in various parts of the body, including the prostate [3], liver [4], pancreas [5], breast [2,6], kidney [4,7], uterus [8], brain [9] and bone [10]. FUS operates by focusing ultrasound waves on a target region, inducing rapid compressions and expansions within the tissue. When used for ablation, this process generates localized heating due to the thermo-viscous properties of soft biological tissues; this heating can result in temperatures above 60 °C [11]. These elevated temperatures cause protein denaturation and irreversible tissue necrosis [12–14]. FUS’s precision in delivering energy to small focal targets (typically 1–3 mm in width and 10 mm in length [1,12]) allows for targeted ablation while minimizing damage to surrounding healthy tissues.

FUS offers several advantages over traditional treatments, including reduced pain [12,15], faster recovery times [16], and the absence of ionizing radiation, making it suitable for repeated use [1]. Furthermore, it provides superior penetration and energy control compared to other thermal therapy heating sources such as lasers, microwaves, and radiofrequency fields [17]. However, challenges remain. The need for inter-sonication cooling periods to protect surrounding tissues extends treatment times [18,19]. Sessions can last up to two hours for 2–3 cm tumors [1], and factors such as organ movement, phase aberration from heterogeneous tissues [20], and difficulties in temperature monitoring in and near adipose tissues [2,19] further complicate treatment. Additionally, poor acoustic coupling may cause undesirable side effects such as skin burns [21]. These challenges highlight the need for and potential of optimized treatment protocols.

Computational predictive models [11,13,17,22–25] that combine acoustic and thermal simulations could be used to enhance FUS treatment planning. These models often rely on the Pennes Bioheat Transfer Equation (PBTE) [26] to simulate temperature distributions based on power deposition fields derived from simulations of the FUS acoustic pressure. Validated models [19,27–29] have shown promise in enhancing treatment accuracy [17,30], reducing damage to healthy tissue [31], and decreasing procedure times [32]. However, many existing models assume constant thermal and acoustic properties, which may oversimplify the tissue response during FUS ablation. Experimental evidence suggests that properties such as thermal conductivity (k), specific heat capacity ($c_{p}$), perfusion (ω), acoustic attenuation (α), and speed of sound (c) [14,33–36] vary significantly with temperature, potentially impacting the accuracy of predictions.

Previous studies have explored the influence of temperature-dependent thermal and acoustic properties in FUS computational modeling. One pilot and follow up study incorporated temperature-dependent thermal conductivity (k) and specific heat capacity ($c_{p}$) in *ex vivo* liver tissue models [37,38], revealing that constant-property models overestimate lesion size by neglecting dynamic thermal behavior. Another study implemented temperature-dependent acoustic properties along with dynamic perfusion modeling in hyperthermic liver treatments, demonstrating that variations in acoustic absorption are the primary factor influencing power deposition and thermal dose predictions [20]. These prior works have primarily focused on homogeneous *ex vivo* tissues, with perfusion effects considered only in hyperthermic regions, and have not systematically evaluated the frequency at which temperature-dependent properties should be updated in explicit numerical simulations.

This study extends previous research by systematically incorporating temperature-dependent thermal properties into computational models for FUS treatments, explicitly examining three distinct scenarios. First, we utilize a homogeneous, *ex vivo* liver model to investigate how the frequency of updating temperature-dependent properties influences the accuracy and computational cost of simulations, an aspect not systematically addressed in prior literature. Additionally, this scenario evaluates how the selection of baseline temperatures for constant-property models impacts the accuracy of thermal and necrosis predictions. Second, we expand the analysis to heterogeneous, *in vivo* rabbit thigh muscle models and validate against experimental magnetic resonance temperature imaging (MRTI). Finally, we introduce a scenario using the same heterogeneous rabbit model but incorporating temperature-dependent acoustic properties, examining their effect on power deposition and heating patterns, as well as assessing how the frequency of acoustic property updates affects simulation accuracy. These analyses collectively provide critical insights for refining simulation-based treatment planning.

## Methods

### Simulation scenarios

The study included explicit finite-difference time-domain (FDTD) simulations in three scenarios: (1) a homogeneous liver model with temperature-dependent thermal properties, (2) an *in vivo* rabbit thigh muscle model with temperature-dependent thermal properties validated against experimental MRTI data [39], and (3) an extended model of the rabbit data incorporating both temperature-dependent acoustic and thermal properties. The liver model was chosen because liver is a promising FUS target, has relatively high perfusion, and its temperature-dependent thermal properties are well characterized, while the rabbit model was chosen as a more complex heterogeneous domain where accurate MRTI can be performed for experimental validation.

In all scenarios, both constant-property and temperature-dependent property models were implemented to compare their effects on FUS-induced heating and necrosis prediction.

To reduce computational costs, this study evaluated the impact of updating temperature-dependent properties at different intervals. A systematic time sensitivity analysis determined whether updating properties at every time step is necessary or if longer update intervals can maintain accuracy while enhancing computational performance.

### Scenario 1: liver tissue model

**Figure 1.**
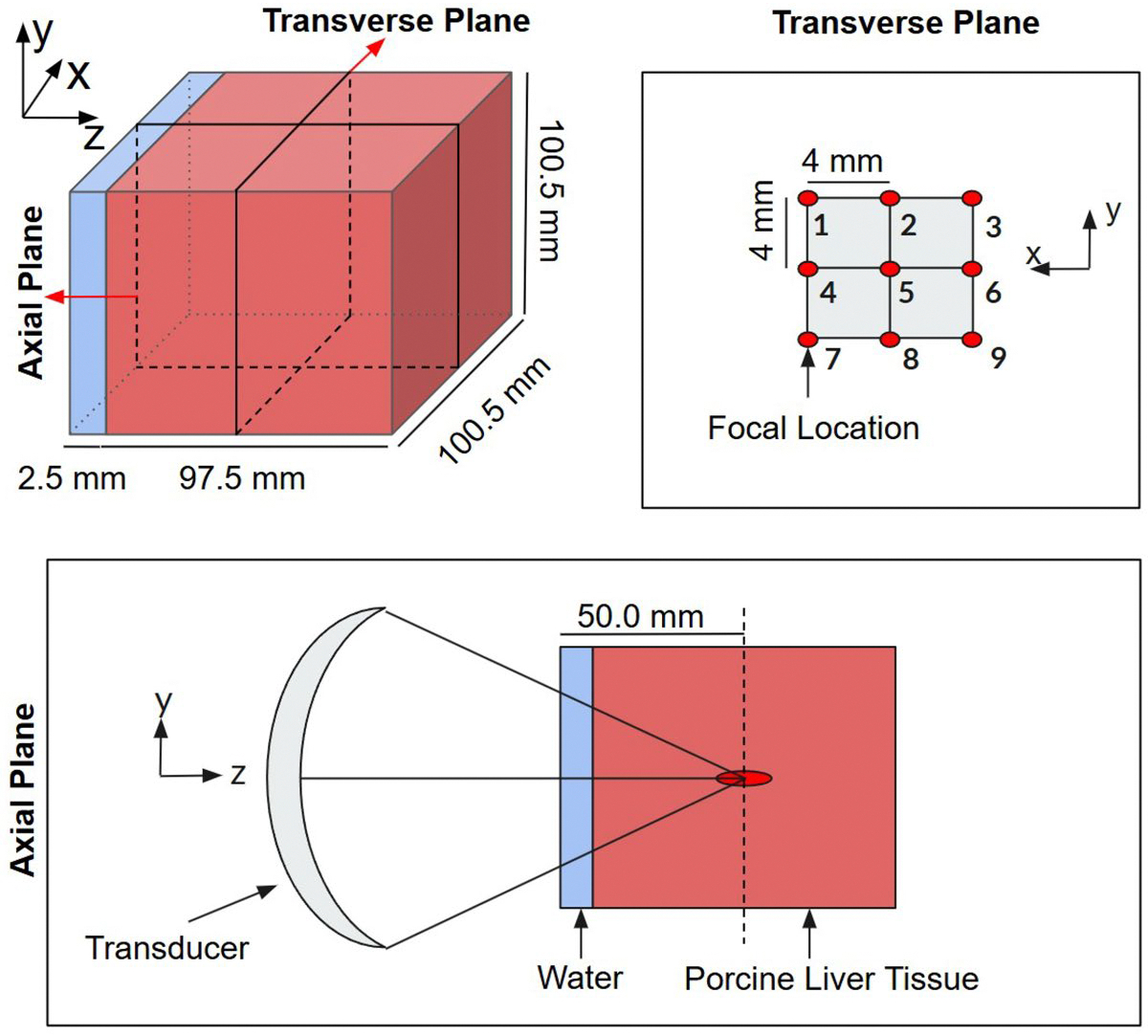
Schematic representation of the 3D liver block model used in fuS simulations.

In the first scenario, liver tissue simulations were conducted using a homogeneous model consisting of a porcine liver block submerged in water for acoustic coupling discretized with 0.5-mm isotropic spacing. Nine FUS sonication focal points were arranged in a 3 × 3 grid, with each point separated by 4 mm. This spacing was selected based on the FUS beam width (pressure full width at half maximum ~2.0–2.5 mm) so that thermal diffusion would generate complete ablation coverage of the region for high power sonications. Figure 1 illustrates the full liver model (top-left), the focal locations in the transverse plane (top-right), and the axial plane depicting the transducer, water coupling layer, and focal region within the liver (bottom).

Three cases were simulated in the liver tissue model to evaluate how variations in power intensity and heating duration affect heating patterns and model response. Case 1 applied a high power 60 W for 30 s per focal location with 30 s of cooling between each sonication, simulating an aggressive ablative FUS treatment where perfusion shutdown from coagulative necrosis is anticipated [40]. Case 2 decreased the power to a more moderate 30 W with the same heating and cooling durations. Case 3 explored mild hyperthermia by simultaneously heating all focal points at 3.3 W for 10 min, simulating rapid electronic steering conditions relevant for FUS drug delivery enhancement [41].

### Scenario 2: rabbit muscle models

The second scenario was based on two *in vivo* rabbit experiments with FUS heating in the thigh muscle. During these experiments, MRTI was performed in a 3 T Siemens Prisma^FIT^ MRI scanner (Erlangen, Germany) to monitor temperature changes in the muscle using the proton resonance frequency shift method [42] with a custom-built single-channel loop radiofrequency coil and the following MRI sequence parameters: 3D gradient echo with segmented echo planar imaging readout, repetition time/echo time = 25/13 ms, bandwidth = 1000 Hz/pixel, flip angle = 14°, echo train length = 7, matrix size = 192×256, 10 slices, voxel size = 1.5×1.5×2 mm^3^. Rabbit 1 received four individual sonications with transducer powers of 48.3, 42.2, 42.2, and 41.8 W applied at different spatial locations in the muscle. Rabbit 2, in contrast, underwent five lower-power sonications of 8.8, 16.0, 16.0, 16.0, and 16.3 W. Additional details of the experiments are described in [39].

Each sonication lasted for 33.6 s in Rabbit 1 and 30.2 s in Rabbit 2, with a cooling period exceeding five minutes between sonications. This cooling period ensured that cumulative heating effects were minimized and enables each sonication to be treated independently.

**Figure 2.**
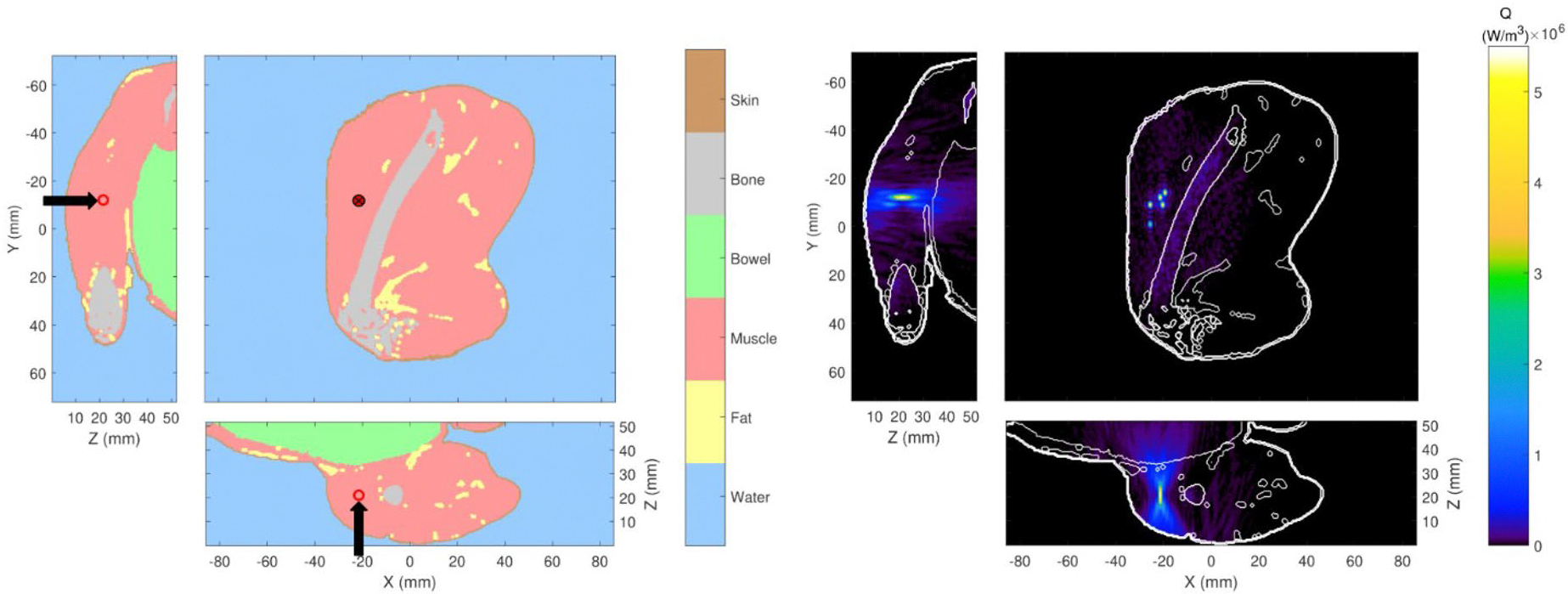
Segmented anatomical model of rabbit 2 (left) and corresponding power deposition distribution from five superimposed sonications (right). arrows in the left panel indicate the transducer’s orientation and focal location for Sonication 5.

For rabbit simulations, the computational models were generated from MRI-based segmentation of the experimental data. Zero-filled interpolation was utilized to adapt the MRI scans to 0.5-mm isotropic spatial resolution [43]. The left panel of Figure 2 shows the segmented model of Rabbit 2, highlighting the different types of tissue and their positions within the computational domain across three orthogonal planes. Each voxel was assigned a tissue type based on the segmented anatomical structures, including muscle, fat, skin, bone, bowel, and water-filled regions. Arrows in Figure 2 indicate the FUS path direction and circles mark the focal location for Sonication 5. The right panel of Figure 2 displays the simulated acoustic power deposition superimposed for all five sonications. More details of the acoustic simulations are provided later in the Methods section.

### Scenario 3: temperature-dependent acoustic properties

Previous sensitivity studies using constant tissue properties have found that acoustic properties have a larger impact on FUS outcomes than thermal properties [20,28]. However, the increased complexity and computation time necessary for coupling acoustic and thermal simulations could be prohibitive. To begin investigating this, the third scenario explored the effect of temperature-dependent *acoustic* properties on computational predictions of a single sonication in the rabbit thigh muscle. By coupling temperature-dependent acoustic properties with temperature-dependent thermal properties, this scenario assessed how variations in acoustic attenuation and speed of sound influence power deposition patterns, temperature distributions, and necrosis predictions as well as the computational cost of those improvements.

Across all scenarios, constant-property models assumed fixed thermal and acoustic properties throughout the simulations, while temperature-dependent models dynamically updated these properties as a function of local tissue temperature. How the property models are implemented is described below.

### Acoustic and thermal simulations

The computational framework for this study integrates acoustic simulations to determine the FUS power deposition within the tissue, followed by thermal simulations using the PBTE [26] to predict the temperature distribution and necrosis volume.

### Acoustic simulations

The Hybrid Angular Spectrum (HAS) method was employed to calculate the acoustic pressure distributions generated by a 256-element phased-array FUS transducer with 10-cm radius of curvature and operating frequency of 940 kHz (Image Guided Therapy, Pessac, France, and Imasonic, Voray-sur-l’Ognon, France). The HAS method was chosen for its computational efficiency and validated accuracy, with deviations of less than 4% compared to the k-Wave method [28,39]. The HAS method computes acoustic wave propagation in inhomogeneous media, accounting for variations in tissue properties including density, speed of sound, and acoustic attenuation.

The acoustic pressure fields generated by the HAS method were used to compute power deposition (Q_FUS_) within the tissue using established relationships between acoustic pressure, absorption coefficient, density, and speed of sound [31,39]. Q_FUS_ serves as the heat source in subsequent thermal simulations.

In scenarios with constant acoustic properties (Scenario 1 and Scenario 2), the pressure patterns and Q_FUS_ were computed at the beginning of the simulation and remained fixed throughout the heating. When acoustic properties were temperature-dependent (Scenario 3), the global pressure patterns and corresponding power deposition maps were recomputed periodically to account for changes in attenuation and speed of sound due to local temperature variations.

### Thermal simulations

The PBTE [26] defined by Equation 1 predicted the tissue temperature distribution T (°C) as a function of time t (s).


(1) $$\rho c_{p}\frac{\partial \;T}{\partial t}=\nabla \cdot (k\nabla \;T)-\omega c_{b}\left(\;T-T_{a}\right)+Q_{FUS}+Q_{m}$$

In Equation 1, ρ (kg/m^3^), $c_{p}$ (J/kg°C), and k (W/m°C) are the density, specific heat capacity, and thermal conductivity of the tissue, $c_{b}$ (3617 J/kg°C [44]) is the specific heat capacity of the blood, ω (kg/m^3^s) is the perfusion rate, $T_{a}$ (37 °C) is the constant arterial blood temperature, and $Q_{FUS}$ (W/m^3^) and $Q_{m}$ (W/m^3^) are the power generated by the focused ultrasound and metabolic processes, respectively. The PBTE incorporates heat diffusion, metabolic heat generation, energy deposition from FUS, and its simple accounting for physiological cooling in perfused tissues makes it a commonly applied model for *in vivo* thermal simulations. Because $Q_{m}$ is orders of magnitude smaller than $Q_{FUS}$, it was neglected in this study [45].

The PBTE was solved using an explicit finite-difference time-domain (FDTD) method. As mentioned previously, each of the tissue models were discretized with an isotropic spatial resolution of 0.5 mm. This resolution effectively captures localized FUS heating patterns while maintaining practical computational costs, as demonstrated in previous studies [46,47]. Time steps of 0.1 s were implemented for liver simulations and 0.05 s for rabbit tissue simulations to ensure numerical stability. The output of the PBTE provided temperature distributions throughout the tissue, which were subsequently used to predict tissue necrosis.

### Necrosis predictions

TD, with units of cumulative equivalent minutes at 43 °C (CEM43), is a quantitative metric used to predict tissue necrosis [48]. It represents the combined effects of elevated temperatures and heating time on tissues and is defined in Equation 2.


(2) $$TD=\int_{t_{0}}^{t_{1}}R^{\left(T-43\right)}dt$$

In Equation 2, R is a dimensionless constant with values of R=4 for 37°C<T<43°C and R=2 for T≥43°C. The start and end time, in minutes, of the treatment are $t_{0}$ and $t_{1}$ respectively. The TD threshold of necrosis for most soft tissues ranges from 25 to 240 CEM43 [22], with 240 CEM43 used as the necrosis threshold for this study.

### Tissue properties and computational implementation

A tissue properties database [44] provided the properties for most tissue types, which are presented in Table 1. In each simulation, properties for water, fat, bowel, bone, and skin were kept constant, because heating was minimal in those tissues and, in many cases, temperature-dependent data are scarce. For all tissues and scenarios, density values were held constant, as previous studies have shown that variations in density have a negligible impact on simulation outcomes [20,49].

### Liver and muscle acoustic properties

For liver tissue in Scenario 1, the speed of sound and acoustic attenuation were retrieved from a tissue properties database [44]. These properties were assumed constant, and the acoustic absorption was assumed to be equal to the attenuation coefficient [37,47,50].

For rabbit muscle tissue in Scenario 2, the speed of sound and acoustic attenuation values were measured directly and reported previously [39]. Additionally, the authors identified acoustic absorption values of 54.1% and 52.7% of the total attenuation that aligned with MRTI data for Rabbit 1 and Rabbit 2, respectively.

In Scenario 3, temperature-dependent acoustic properties were dynamically updated using porcine muscle tissue attenuation values from the literature [51]. The temperature-dependent attenuation in the literature at 0.5 and 1.6 MHz was interpolated to this study’s 0.94 MHz frequency, then scaled to match the experimentally determined acoustic attenuation of Rabbit 1 at 20 °C [39]. In essence, this approach utilized the shape of the temperature-dependent curve in the literature with the baseline attenuation measured in the actual tissue specimen.

### Liver and muscle thermal properties

For liver [35] and muscle tissue [51], temperature-dependent thermal conductivity (k) and specific heat capacity ($c_{p}$) were obtained from curve-fitted functions reported in the literature. Those functions (plotted in the Supplemental Material) were applied dynamically based on the local temperature at each voxel.

In the constant-property models, values for k and $c_{p}$ were taken from the curve-fitted functions at specific temperatures: 25 °C and 37 °C for liver tissue, and 20 °C and 37 °C for rabbit muscle tissue. Some studies in the literature report properties measured at room temperature and others at body temperature, while other studies do not report the tissue temperature at which measurements were made. We wanted to investigate the impact of choosing constant properties measured at room temperature versus using constant properties that closely match the *in vivo* tissue temperature, approximately 37 °C.

For temperature-dependent simulations, a dynamic perfusion (ω) model was implemented by multiplying the baseline perfusion value from the literature [44] by a relative perfusion factor described in [32,52] and defined by Equation 3. This model was applied to the liver and rabbit muscle tissue where the FUS heating was localized. All other tissues retained constant perfusion values based on literature-reported data.


(3) $$\omega_{\mathrm{rel}}(T)=\left\{\begin{array}{cc} 1 & T\leq 37^{\circ }C\\ 0.12T-3.44 & 37<T\leq 42\\ 9.79-0.195T & 42<T\leq 46\\ 3.12-0.05T & 46<T\leq 60\\ 0 & T>60 \end{array}\right.$$

As demonstrated by Equation 3, the perfusion model accounts for hyperemia (increased perfusion) due to moderate heating with a peak at 42 °C and includes full perfusion shutdown (due to vascular damage) at temperatures exceeding 60 °C. In addition to perfusion’s temperature-dependence, an irreversible shutdown condition was implemented: once the local tissue temperature reached or exceeded 60 °C, its perfusion was set to zero for the remainder of the simulation, even if the local temperature later decreased. This condition reflects irreversible vascular damage observed in biological tissues and improves the accuracy of the thermal response modeling.

### Implementation of temperature-dependent properties

In Scenario 1 and Scenario 2, acoustic pressures and power deposition patterns were calculated only once at the beginning of the simulation. For thermal simulations utilizing temperature-dependent properties, updates occurred dynamically. After each time step of the explicit FDTD solver of the PBTE, the temperature distribution was used to update the thermal conductivity (k), specific heat capacity ($c_{p}$), and perfusion (ω) of the liver or muscle tissue based on the local temperature at that time.

After each FDTD time step of Scenario 3, which accounts for temperature-dependent acoustic properties, local temperatures were used to update acoustic attenuation and speed of sound, and the acoustic pressure and power deposition patterns from HAS were recomputed. Thermal properties were also updated dynamically as described above. Thus, the simulations of Scenario 3 evaluated the impact of temperature variations on both acoustic and thermal properties throughout the heating process.

To improve computational efficiency, precomputed lookup tables were generated to store property values for temperatures ranging from 37 °C to 90 °C in 0.1 °C increments. This approach provided a smooth and precise estimate of property values, while greatly reducing computational overhead compared to direct evaluation of temperature-dependent functions at runtime.

### Time sensitivity analysis

Temperature-dependent thermal simulations in Scenario 1 and Scenario 2 were repeated several times, with thermal properties updated at varying time intervals (i.e., not every FDTD timestep) to assess how the update interval affected temperature predictions and necrosis volumes. In liver simulations, update intervals ranging from 0.1 to 15.0 s were tested, while in rabbit simulations, intervals from 0.05 to 10.0 s were evaluated. Based on preliminary findings, a thermal property update interval of 1.25 s was chosen for Scenario 2 as it provided accurate results while reducing computation time. Details supporting this choice are presented in the Results section.

In Scenario 3, time sensitivity tests were performed in two sequential steps. First, thermal properties were updated at intervals of 0.05 and 1.25 s, while acoustic properties were updated every FDTD time step of 0.05 s. This step confirmed that a 1.25-s thermal property update interval maintained accuracy and precision. Based on this, the second step fixed the thermal property update interval at 1.25 s while varying the acoustic property update interval from every time step of 0.05 s to 10.0 s.

### Temperature-dependence of perfusion versus other properties

To distinguish which temperature-dependencies were most critical, two additional simulations were performed for Scenario 1, in Cases 1 (60 W) and 2 (30 W). In the first simulation, perfusion ω was allowed to vary with temperature, and specific heat capacity $c_{p}$ and thermal conductivity k were fixed with constant values at 37 °C. In the second simulation, specific heat capacity $c_{p}$ and thermal conductivity k varied with temperature while the perfusion ω was held constant. This second simulation is similar to the protocol utilized by Guntur and Choi [37,38].

### Computational environment

All simulations were conducted using MATLAB 2023b [53] on a system equipped with an Intel(R) Core(TM) i7–10750H CPU @ 2.60 GHz and 32.0 GB of RAM.

## Results

### Scenario 1: liver tissue High- and moderate-power ablation: Cases 1 and 2

**Figure 3.**
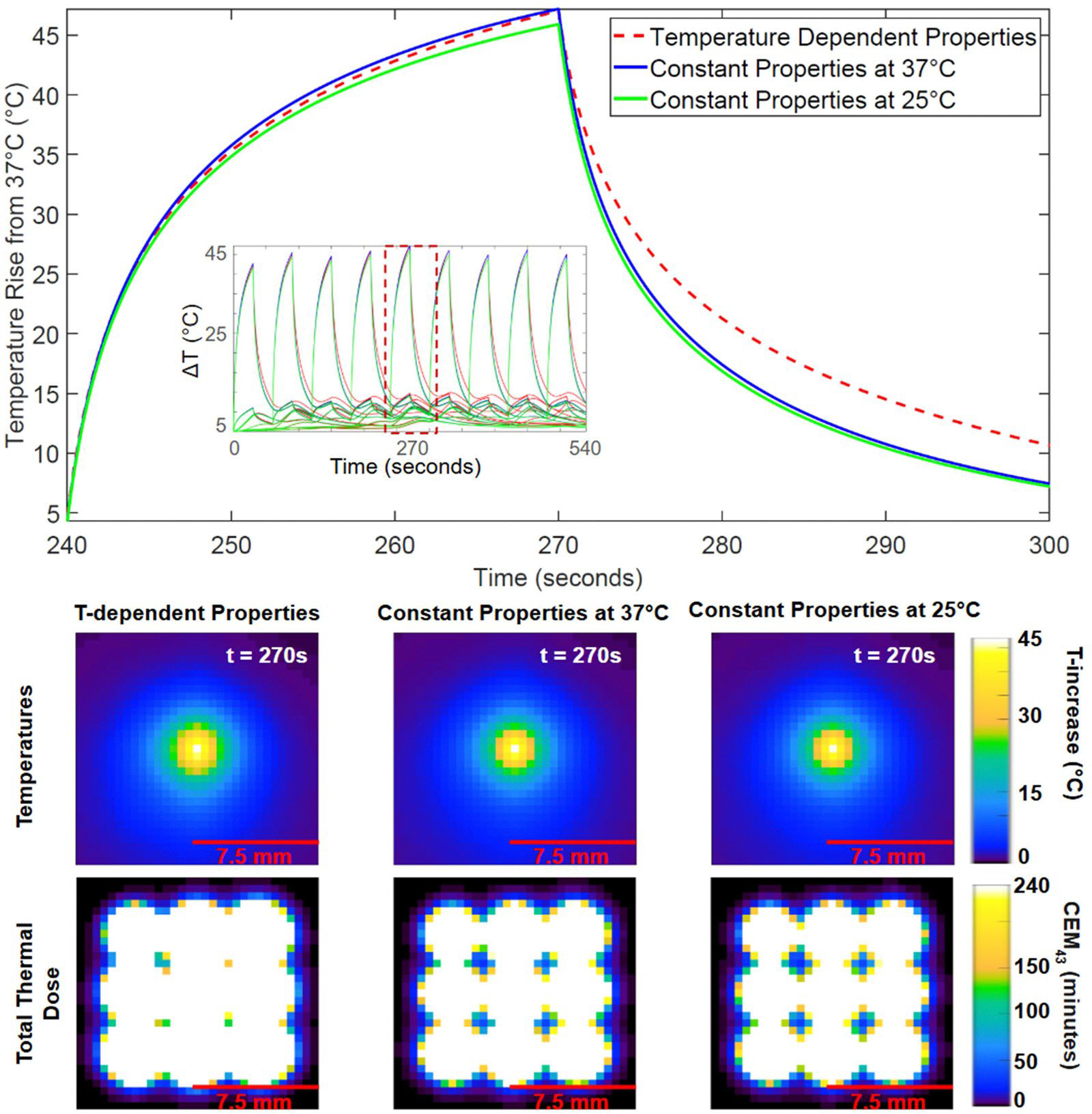
Temperature rise from 37 °C and thermal dose distribution for Case 1 (60 W) in the liver tissue model.

Figure 3 highlights results from the liver tissue simulations for Case 1, where a transducer power of 60 W was applied for 30 s to each focal point with 30 s of cooling between sonications. The top plot of Figure 3 presents the temperature increase versus time at the center of Sonication 5 during its heating and cooling period (240–300 s) for the temperature-dependent model and the two constant-property models (37 °C and 25 °C). The inset graph in that top plot shows the temperature evolution above 37 °C for all nine focal locations, with the dashed line box indicating the period for the larger plot. The temperature increase for each of the three property models was approximately 46 °C, with variation between models less than 1.12 °C. Notably, the temperature-dependent model exhibited a slower temperature decrease during cooling, consistent with perfusion shutdown caused by the large temperature rise.

The middle row of Figure 3 displays the spatial temperature increase distribution in the transverse plane at 270 s (at the end of the heating period for sonication 5) for each of the different property models. The bottom row shows the thermal dose map in the transverse plane for each property model. The total necrosis volume for Case 1 was 1466 mm^3^ for the temperature-dependent tissue property model. Constant property models predicted necrosis volumes 18% smaller for properties at 25 °C and 13% smaller for properties at 37 °C.

Case 2 of Scenario 1 applied a more moderate power of 30 W to the liver tissue, but with other parameters unchanged from Case 1. Plotted highlights of Case 2 are included in the Supplemental Material, Figure S8. Compared with the high-power simulations of Case 1, Case 2 had less overall variation in temperatures and necrosis volume between models. The observed difference between models in cooling temperature profiles during cooling (Figure 3, top plot) was not present in Case 2, because the temperature did not increase sufficiently to initiate perfusion shutdown in the simulations.

Table 2 compares maximum temperature increases, necrosis volumes, and computation times for each model for Case 1 and Case 2. The temperature-dependent models required a factor of four increase in computation time due to dynamic property updates.

**Figure 4.**
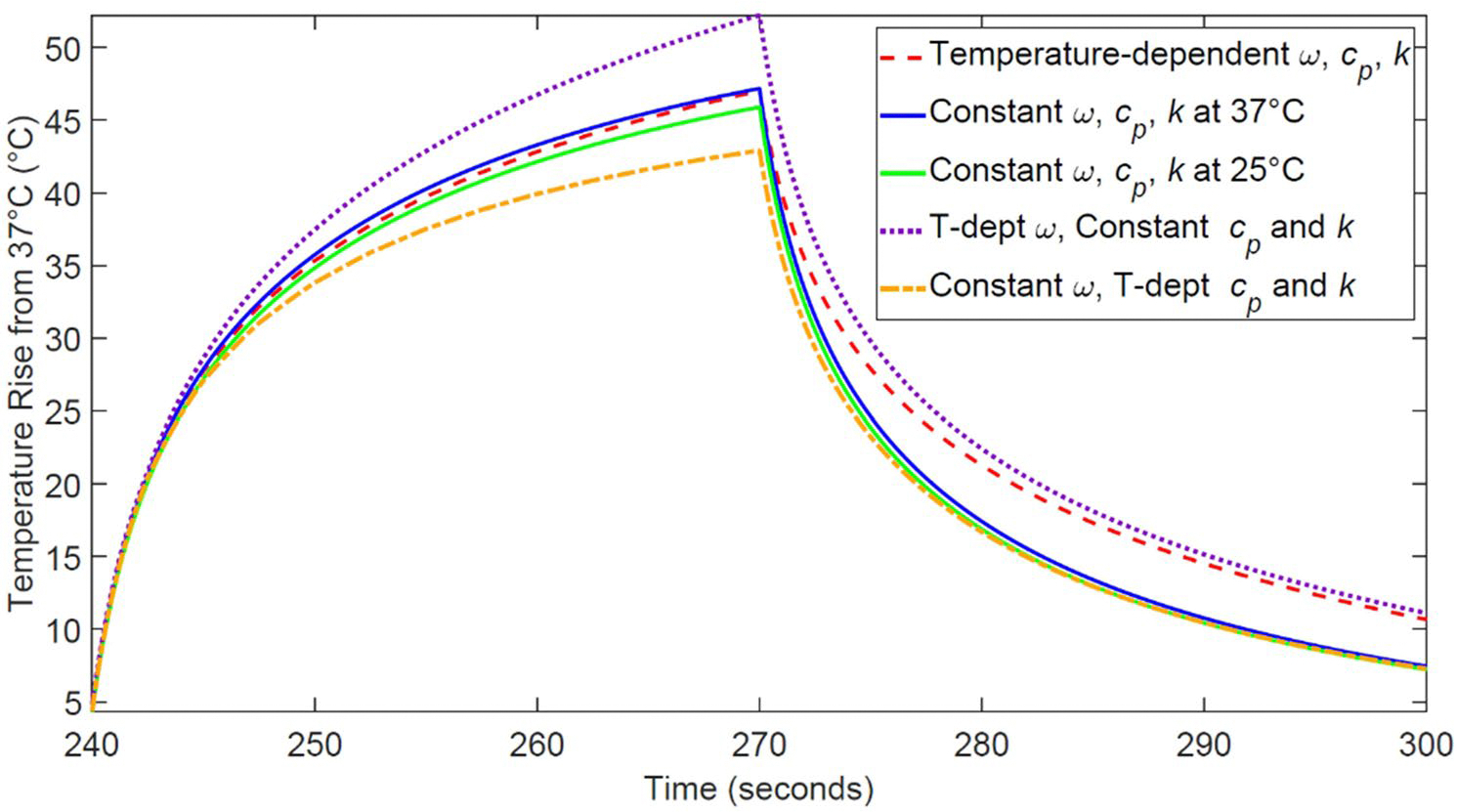
Temperature rise from 37 °C versus time for Scenario 1, Case 1 (60 W) utilizing various combinations of temperature-dependent and constant property models for perfusion ω, specific heat capacity cp, and thermal conductivity k.

Figure 4 differentiates the effects of temperature-dependent perfusion ω from those of specific heat capacity $c_{p}$ and thermal conductivity k in Scenario 1, Case 1 (60 W). It includes the temperature-versus-time profiles of the top plot of Figure 3 and two more simulations using different combinations of constant and temperature-dependent property models. Temperature-dependent ω with constant $c_{p}$ and k (dotted purple line in Figure 4) led to an increased temperature rise of 52.18 °C with a 1509 mm^3^ total volume of necrotized tissue. Constant ω with temperature-dependent $c_{p}$ and k (dashed-dotted orange line) led to a decreased temperature rise of 42.92 °C with a necrotized tissue volume of 1223 mm^3^.

### Time sensitivity analysis

Table 3 shows the impact of different thermal property update intervals for the temperature-dependent model in Case 1 (60 W) and Case 2 (30 W). Changes to the update interval had minimal impact (<1% difference) on the maximum temperature change of the simulations. As the updated interval increased, differences in predictions of necrosis volume were initially small then grew as large as 8.1% with an update interval of 15 s. Importantly, computation time decreased sharply as the update interval increased, with the greatest reduction occurring between 0.1 and 2.5 s. Updating thermal properties every 2.5 s (highlighted row of Table 3) produced temperature rise and necrosis predictions comparable to updates at every 0.1 s, while reducing computation time by 68%.

### Mild hyperthermia: Case 3

**Figure 5.**
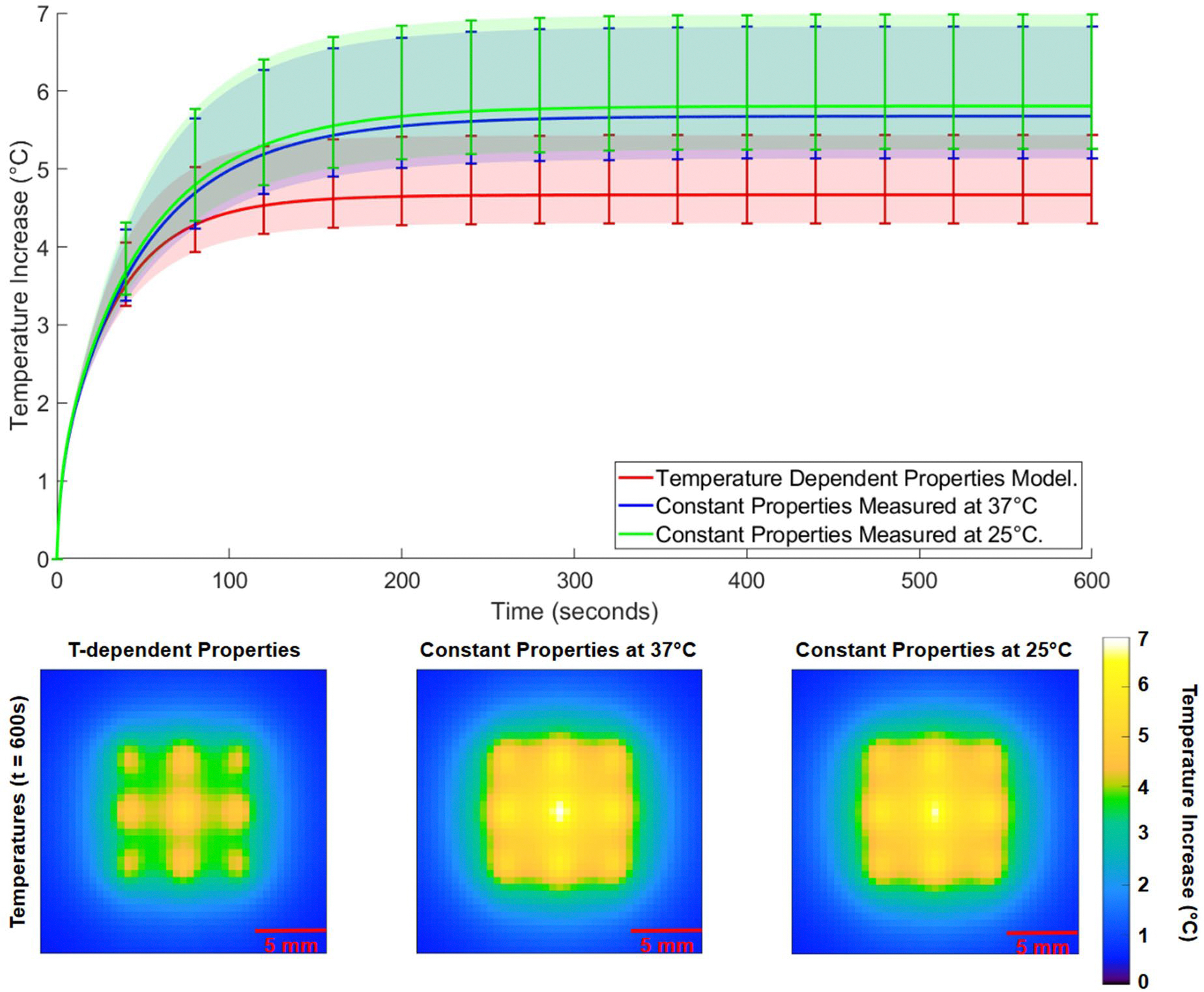
Temperature evolution and spatial distribution in the liver tissue model under mild hyperthermia conditions (Case 3, 3.3 W per focal point). Increases occur from a baseline initial temperature of 37 °C.

Figure 5 shows the temperature evolution and spatial distribution in the liver tissue model under mild hyperthermia conditions, where a transducer power of 3.3 W was applied simultaneously to all nine focal points for 600 s. The top panel of Figure 5 presents the average (solid line) and range (shaded region with bars) of temperature increases across the nine focal points for the temperature-dependent model and the two constant-property models (25 °C and 37 °C). The temperature-dependent model (red) exhibited a smaller average and range of temperature increase than the constant property models. The average temperature at the end of the simulation was 5.8 °C for the constant property model at 25 °C (green), 5.7 °C for the constant property model at 37 °C (blue), and 4.7 °C for the temperature-dependent model (red).

The bottom panel of Figure 5 illustrates the temperature distribution in the transverse plane at 600 s. The cooler temperature distribution observed in the temperature-dependent property model is a result of relative perfusion increases at hyperthermic temperatures defined in Equation 3.

### Scenario 2: rabbit models

#### Time sensitivity analysis

The time sensitivity analysis for Scenario 2 is detailed in the Supplemental Material. Consistent with Scenario 1 results, this analysis demonstrated that updating properties every 2.5 s rather than every time step (0.05 s) enabled substantial reductions in computation time (63% reduction in computation time) without compromising accuracy in predicting temperatures and necrosis volumes.

#### Temperature and thermal property distributions

**Figure 6.**
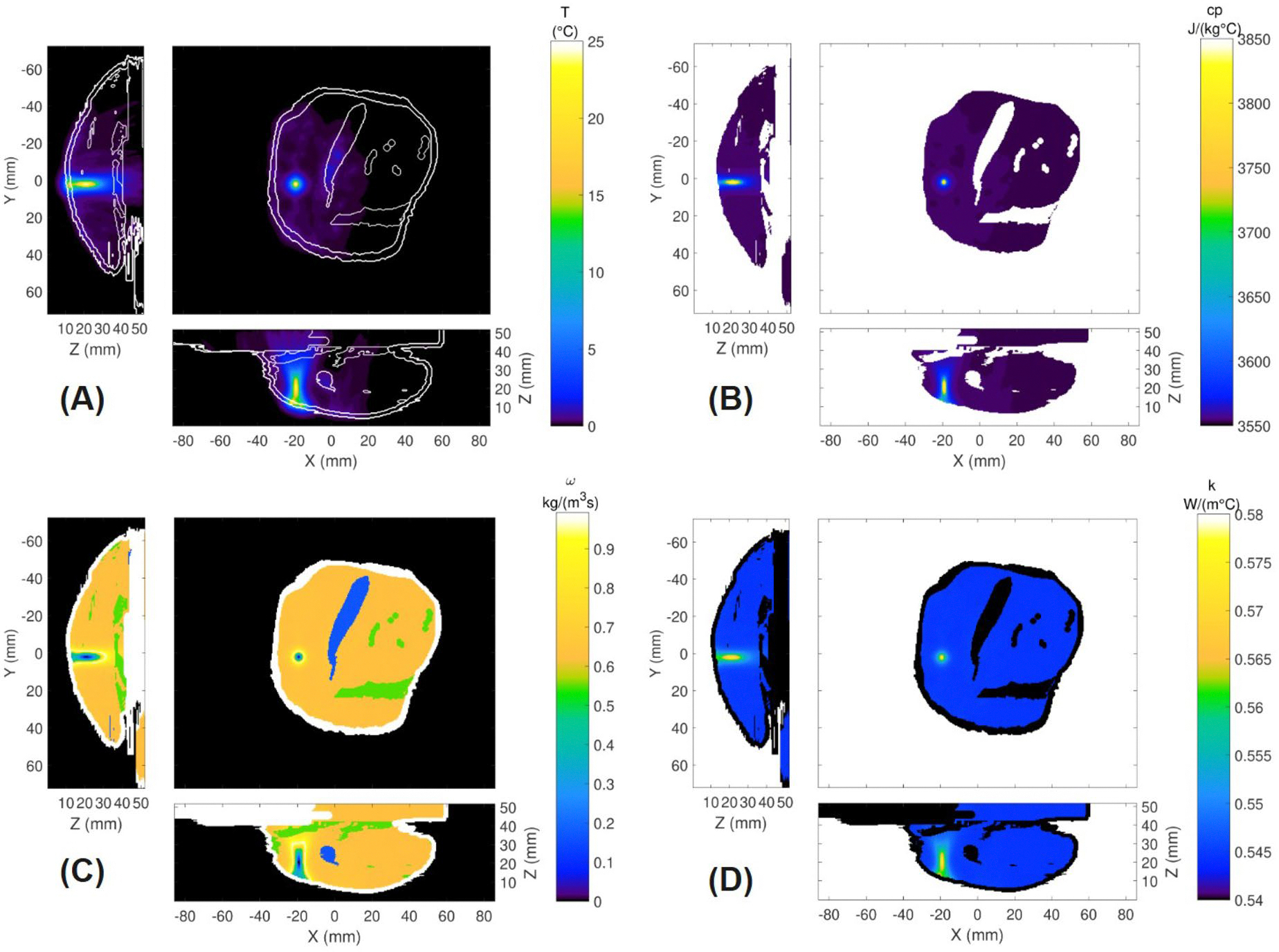
Spatial distributions for (a) temperature increase at the end of heating for rabbit 1, Sonication 1, (b) Specific heat capacity $c_{p}$, (c) perfusion *ω*, and (d) thermal conductivity *k.*

Figure 6(a) presents the simulated temperature increase at the end of the heating period for Rabbit 1, Sonication 1 using the temperature-dependent property model. Spatial maps of the temperature-dependent properties at that time are also presented for specific heat capacity $c_{p}$ (Figure 6(b)), perfusion ω (Figure 6(c)), and thermal conductivity k (Figure 6(d)). The dynamic property changes are localized to the focal region in this simulation, but would be expected to expand into the near field with a treatment requiring many sonications [54] or when utilizing volumetric heating techniques [55,56]. A second example of the spatial temperature and property distributions is presented in the Supplemental Material for a sonication with a small temperature rise of 6 °C.

### Comparison of simulated and experimental temperature profiles

A table summarizing results from each of the nine sonications in rabbit thigh muscle for Scenario 2 is provided in the Supplemental Material. For these simulations in rabbit muscle, the temperature-dependent models predicted lower temperature rises and a smaller necrosis volume than the constant property models. However, the variations were small, with less than 6% differences in maximum temperature change and less than 5 mm^3^ differences in necrosis volume. The 1.25 s property update interval used for the temperature-dependent property model simulations resulted in ~17% increase in computation time compared with the constant property models.

**Figure 7.**
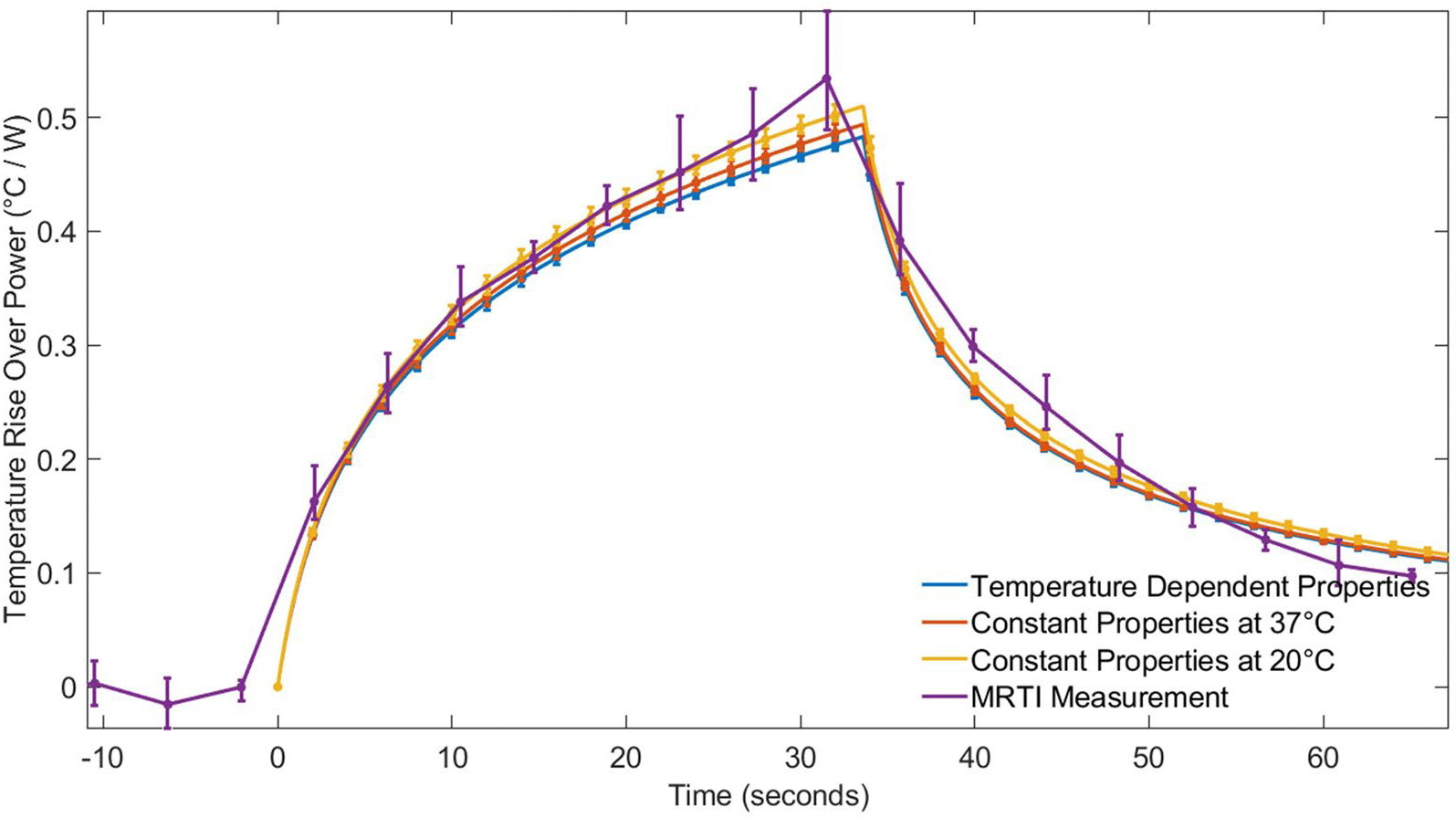
Temperature rise normalized by applied power versus time for the four high power (40–50 W) sonications in rabbit 1.

**Figure 8.**
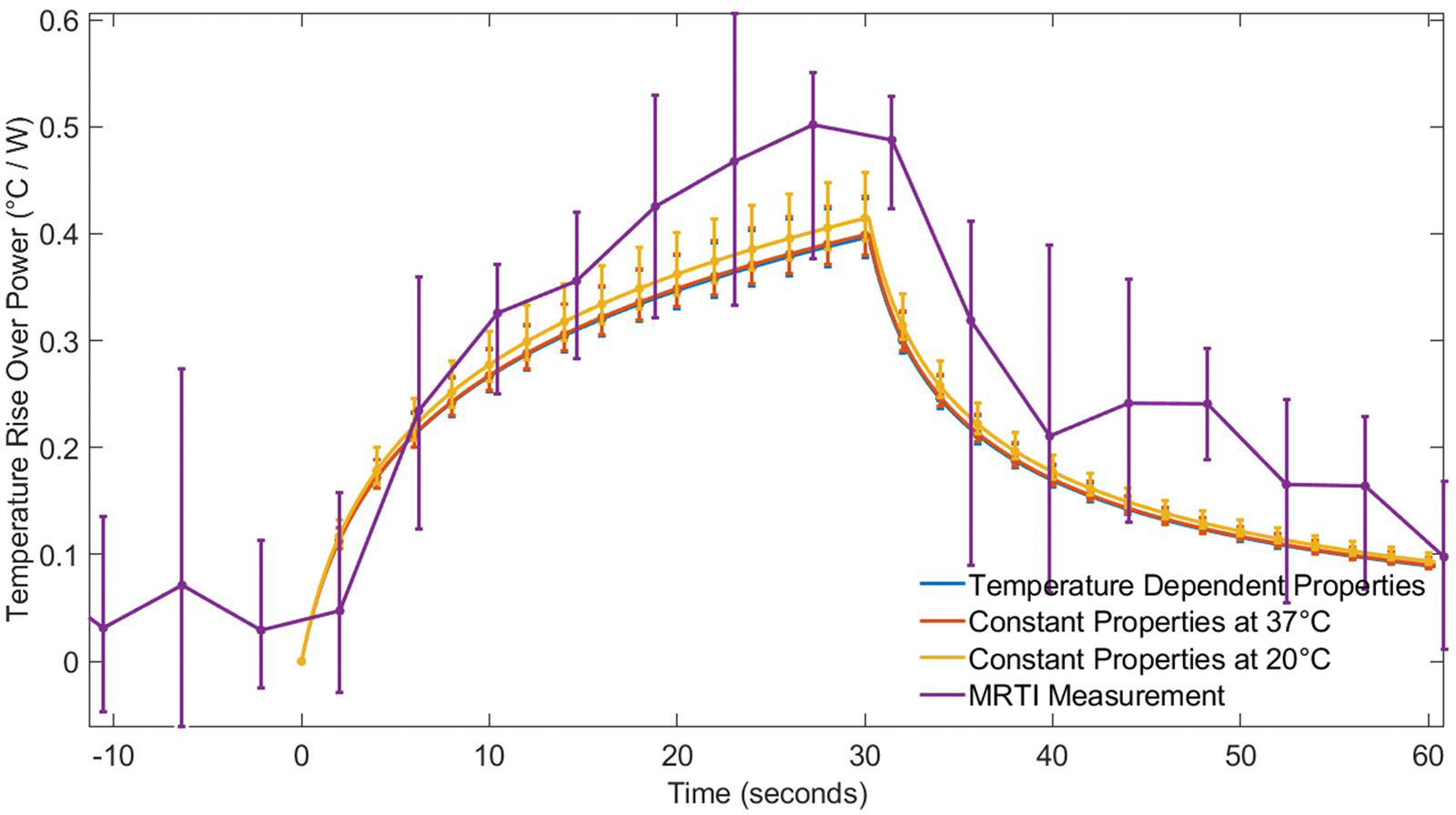
Temperature rise normalized by applied power versus time for the five low power (8–16 W) sonications in rabbit 2.

Figures 7 and 8 show the temperature rise per unit power for all sonications in Rabbit 1 and Rabbit 2, respectively. The temperature rise is normalized by applied power, enabling the comparison of multiple sonications between the experimental MRTI data, the temperature-dependent model, and the constant-property models at 20 °C and 37 °C. Error bars indicate ±1 standard deviation from the mean.

Figure 7 shows that all three property models (constant and temperature-dependent) closely follow the experimental MRTI measurements during the heating and cooling times for moderate to high power sonications in Rabbit 1. The constant-property models at 20 °C and 37 °C consistently show slightly higher temperatures than the temperature-dependent model. As mentioned previously, total variation in the maximum simulated temperature rise was less than 6%. The temperature in Rabbit 1 increased 20–25 °C for the four sonications across all models.

Figure 8 shows the low power simulations for Rabbit 2 were less consistent with MRTI measurements. There is greater variability in the MRTI measurements for both heating and cooling periods in Rabbit 2 compared to Rabbit 1, likely because the temperature rise for Rabbit 2 was much smaller, approximately 6 °C. The temperature-dependent model matches the constant-property models, with only minor variations between them.

### Scenario 3: temperature-dependent acoustic properties

Scenario 3 extended the simulations for one sonication in the rabbit thigh muscle to include temperature-dependent acoustic properties.

### Thermal property update sensitivity

To address whether thermal property update intervals would impact results for acoustic property updates, two simulations were initially conducted with thermal properties updated every 0.05 s and every 1.25 s, while acoustic properties were updated every timestep of 0.05 s. With a thermal property update interval of 0.05 s, the maximum temperature increased 26.14 °C, and the necrosis volume was 21.25 mm^3^. Updating thermal properties every 1.25 s resulted in a very similar maximum temperature increase of 26.16 °C and necrosis volume of 21.375 mm^3^. While these temperature and necrosis volume differences were negligible, computation time was greatly reduced from 113 min, 25 s to 82 min, 53 s with less frequent thermal property updates. The reduction in computation time is consistent with Scenario 1 and 2 results. The total computation times are much greater than those for Scenarios 1 and 2, because acoustic properties and the power deposition profile were updated and recomputed every time step of the heating period.

### Acoustic property update sensitivity

**Figure 9.**
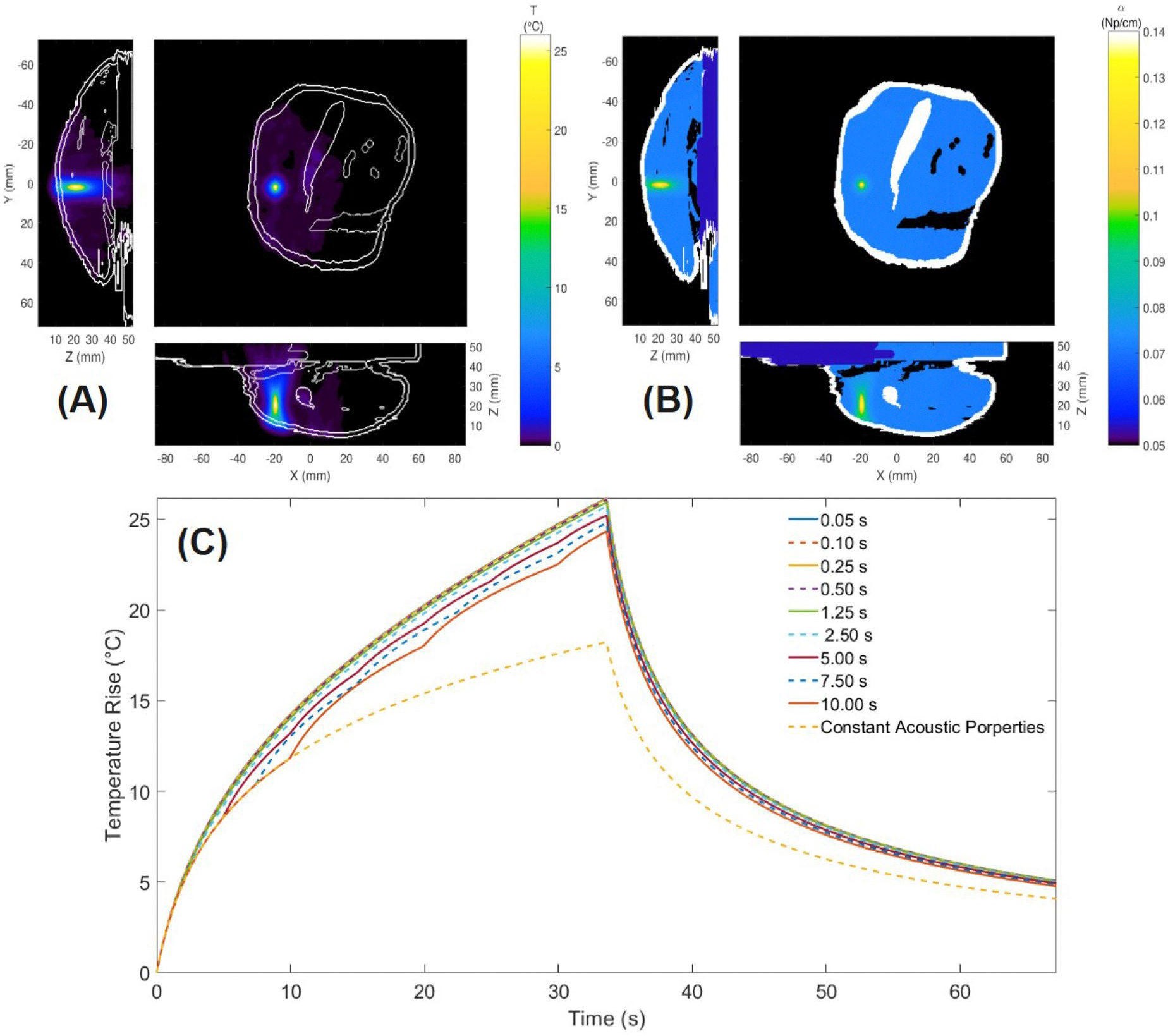
Impacts of temperature-dependent acoustic properties. (a) Spatial temperature distribution at the end of heating and (b) corresponding changes in acoustic attenuation. (c) temperature rise versus time for different acoustic property update intervals.

Figure 9 presents the impact of temperature-dependent acoustic properties and thermal properties updated every 1.25 s. Figure 9(a) shows the spatial temperature distribution at the end of heating with acoustic properties were updated every timestep (0.05 s), and Figure 9(b) presents the corresponding spatial distribution of acoustic attenuation. Hotter regions exhibited higher attenuation. This resulted in greater temperature increases for simulations with more frequent acoustic property updates, as demonstrated in Figure 9(c).

Table 4 compares the maximum temperature rise and necrosis volume for simulations with each acoustic property update interval. Updating the acoustic properties every 1.25 s instead of every 0.05 s changed the predicted temperature rise and necrosis volume by less than 5%, while reducing computation time by almost 90%. As the update interval increased to 10 s, the maximum temperature prediction was almost 2 °C different and the necrosis volume dropped by 27% to 15.500 mm^3^ with smaller improvements in computation time. With constant acoustic property changes, the maximum temperature and necrosis volume predictions were 30% and 85% lower, respectively, than with fully dynamic acoustic properties. These observations indicate a strong sensitivity to temperature-dependent acoustic properties, with increasing errors and decreasing computational gains for progressively larger update intervals.

## Discussion

### Room- versus body-temperature simulations

It may be convenient to measure tissue properties at room temperature in a lab setting, but that generally represents a 10–15 °C difference from *in vivo* tissue temperatures. For this reason, we investigated the impact of simulating FUS with constant thermal properties measured at room temperature (~20–25 °C) and simulating FUS with constant thermal properties at body temperature (~37 °C). We found that for both liver and muscle tissues experiencing high-, moderate-, and low-power sonications, the predicted temperature profiles, temperature distributions and necrosis volumes (see Figures 3, 5, 7, and 8) were generally quite similar for simulations with room-temperature versus body-temperature constant thermal properties. This is reassuring given the lack of consistency in temperatures at which property data are reported in the literature.

### Constant versus temperature-dependent thermal simulations

At moderate powers, liver simulations (Scenario 1, Case 2) revealed that, while peak temperature differences between constant-property and temperature-dependent models were minimal (less than 0.5 °C), predicted necrosis volumes could still have discrepancies. In this case, the constant property models underestimated or overestimated tissue necrosis by 6–7%, depending on the temperature at which the properties were determined. In the moderate-power, rabbit muscle simulations of Scenario 2, the temperature-dependent thermal property simulations consistently predicted temperature rises smaller than both constant property simulations (see Figures 7 and 8), but those temperature differences were still less than 1.5 °C.

These differences in temperature rise and necrosis volume predictions for moderate-power sonications are on the order of the uncertainty in MRTI measurements and may not be large enough to merit the increased complexity and computational cost of implementing temperature-dependent properties.

The interplay of temperature-dependent property models in the simulations is highlighted in Figure 4. While temperature-dependent perfusion models with perfusion shutdown increased the temperature rise and necrosis volume predictions, temperature-dependent specific heat capacity and thermal conductivity models reduced heating. This last result is consistent with the results of Guntur and Choi in predicting reduced focal temperature rise and thermal lesion area with temperature-dependent liver tissue FUS simulations with constant perfusion [37,38]. In this study, by including temperature-dependent tissue property models with opposing effects, the overall impact of those models is reduced. This mitigating effect may not be present for all combinations of FUS heating times, duty cycles, and power levels, especially if incorporating different temperature-dependent property models for these or other tissue types.

In scenarios with perfusion shutdown in highly perfused tissues (high-power sonications) or where hyperemia is induced (low-power sonications), differences between temperature-dependent and constant property models were still substantial as discussed below.

### Dynamic perfusion in simulations

Perfusion shutdown in high-power liver tissue sonications (Scenario 1, Case 1) explains the temperature differences during the cooling period observed in the top panel of Figure 3. Where tissue temperatures exceeded 60 °C, perfusion dropped to zero in temperature-dependent property simulations, eliminating convective heat dissipation through blood flow. As a result, thermal energy remained in the focal region during cooling, causing sustained higher temperatures and a larger necrosis volume. In contrast, constant property models maintained a static perfusion rate, cooling the tissue faster and resulting in a predicted necrosis volume up to 18% smaller than the temperature-dependent property model.

In low-power liver tissue simulations (Scenario 1, Case 3) where mild hyperthermia is simulated, the temperature rise in the entire focal region of the temperature-dependent model is consistently less than that predicted by the constant property models (see Figure 5). A temperature increase of 4–6 °C increased the perfusion rate by 40–60% according to the temperature-dependent perfusion model of Equation 3, magnifying the perfusion heat sink effect of the Pennes model and curbing the tissue temperature increase. One important clinical implication is that traditional constant property models could underestimate the power necessary to maintain temperatures required for hyperthermia-induced or -enhanced drug release [41].

In the rabbit tissue simulations of Scenario 2, differences between the constant property and temperature-dependent property models were relatively mild. Because the baseline perfusion value for muscle tissue is inherently low, perfusion upregulation from moderate temperature increase and perfusion shutdown from large temperature rises didn’t have large effects on the tissue thermal response.

These findings highlight the importance of incorporating temperature-dependent perfusion in highly perfused tissues, particularly for therapeutic applications where high-power ablation induces perfusion shutdown or where maintaining controlled, moderate temperature elevations is critical.

### Balancing improved simulations with increased computational cost

While implementing temperature-dependent property models more fully reflects the physics and may more accurately predict the thermal tissue response, it requires more complex computational tools that have a higher computational cost. In liver simulations of Scenario 1, Table 2 demonstrated that updating temperature-dependent properties every time-step (0.1 s) led to ~400% increases in computation time. However, Table 3 highlights that with a property update interval of 2.5 s, the increase in computation time was only 25–33% and that the thermal response was nearly identical to simulations that update the thermal properties every timestep. Scenario 2 simulations in rabbit muscle found results consistent with Scenario 1 (see Supplemental Material).

Thus, this study demonstrated that the gains of temperature-dependent property models can be realized without exorbitant increases in computational cost by selecting a good property update interval. This could be particularly useful when rapid, near-real time simulations are needed such as in model predictive filtering for improved MRTI [57].

### MRTI validation of simulations

Results from Scenario 2 in rabbit muscle (Figure 7) showed that FUS simulations align with the overall heating and cooling trends observed in MRTI measurements. For Rabbit 2 (Figure 8), the MRTI data exhibited notable noise at lower temperature ranges, due to a reduced signal-to-noise ratio in MRTI measurements. Noise effects are less pronounced in Rabbit 1 (Figure 7), because the temperature rise is so much larger relative to the noise. Despite these noise effects, all models followed the general heating and cooling patterns observed in MRTI data, with temperature differences between models remaining within a narrow range. Such results add to a growing number of FUS simulation validation studies [19,28,39,58] and provide evidence of their value for pretreatment planning, simulation-enhanced treatment monitoring, and retrospective treatment evaluation.

### Temperature-dependent acoustic simulations

The exploratory Scenario 3 emphasizes the important role of temperature-dependent acoustic properties in predicting tissue heating and necrosis during FUS treatments. While thermal properties had a minor to moderate impact on the rabbit muscle tissue thermal response (see Figures 7 and 8), Figure 9 showed that implementing temperature-dependent acoustic properties generated clinically meaningful increases in temperature rise (30% greater) and necrosis volume prediction (85% greater) compared to constant property models.

These findings are consistent with studies in the literature. Johnson et al. [28] found that acoustic attenuation in homogeneous tissue-mimicking phantoms was a primary driver of uncertainty in peak temperature predictions. In a numeric study, Tan et al. [20] identified the dynamic acoustic absorption coefficient (which along with scattering makes up the acoustic attenuation) as having the greatest influence on temperature and thermal damage of all thermal and acoustic properties. That study found a constant property maximum temperature rise (65.94 °C) that was 19 °C less than with temperature-dependent acoustic attenuation.

The shape of the temperature-versus-time profile also changed when including temperature-dependent acoustic properties, with reduced temporal curvature as the sonication progressed (see Figure 9). In this single sonication, as the temperature increased, so did the local acoustic attenuation, leading to a larger local power deposition that reduced the rate at which the temperature curve bent over from thermal conduction. A similar effect is seen in the rabbit MRTI data (see Figures 7 and 8). This is one indication that the inclusion of well-characterized temperature-dependent acoustic properties could improve FUS simulation accuracy in the future.

Given the added complexity of coupling acoustic and thermal simulations and the potential for enormous increases in computation time for frequent acoustic property updates (see Table 4), researchers might be hesitant to incorporate temperature-dependent acoustic properties in their simulation framework. While this study only examined one sonication of a single scenario for acoustic properties, it provides clear and reassuring evidence that, as with thermal properties, a well-chosen acoustic property update interval can yield the benefits of temperature-dependent modeling with an acceptable computational cost. Further computational gains could be possible if the acoustic pressure and power deposition fields were updated only locally since most of the changes to the acoustic field are contained within regions experiencing significant temperature changes. However, this approach would introduce additional complexity and necessitate the establishment of appropriate thresholds, automated masking of regions for property updates, or adaptive meshing strategies.

### Study limitations

While this study provides valuable insights into temperature-dependent modeling of FUS therapies, several limitations remain. The study simulated only a small treatment volume (nine total sonications) and implemented temperature-dependent properties in a single tissue per simulation. Treating large tumors with dozens to hundreds of sonications along a heterogeneous path can lead to near-field heating [54,56]. Temperature-dependent properties in near-field tissues like subcutaneous fat during long treatments could alter the overall treatment response.

This study was also limited to FUS protocols involving heating times of tens of seconds to minutes with 100% duty cycle powers of 3–60 acoustic watts. Such protocols would include continuous-power hyperthermic and ablative FUS treatments but would not include treatments like histotripsy [59,60] or neuromodulation [61,62] where duty cycles, heating times, and power levels are distinct from those of the current study. In such cases, the optimal property update interval would very likely be different from the 2.5-s update interval identified here. For higher power ablative sonications in this study (60 W) and lower power hyperthermia scenarios (3.3 W), perfusion shutdown at high temperatures and hyperemia, respectively, led to clinically relevant changes in the tissue thermal response. In moderate power scenarios (30 W), the effects of temperature-dependent modeling were less pronounced.

The liver model is uniform, homogeneous and far simpler than an actual image-based patient model. Further, the study assumes uniform tissue composition within each tissue type, which does not fully capture the heterogeneity present in biological tissues. Differences in tissue microstructure, including localized variations in perfusion [23], lipid content [2], or fiber orientation [63], could influence the accuracy of acoustic propagation and thermal responses. Acoustic and thermal property changes driven by edema would be worth investigating. Fortunately, the complexity of the computational domain from tissue heterogeneity does not notably impact the computational cost of acoustic and thermal simulations. The computational cost of both acoustic and thermal simulations does increase as the grid size is refined or the extent of the model is expanded. Trends in the computational cost identified in this study should therefore apply to more complex models and scale with model size.

While temperature-dependent liver tissue properties are reasonably well characterized in the literature [35,64,65], temperature-dependent property data for many other tissues are scarce. For example, it was necessary to scale published temperature-dependent acoustic attenuation of porcine muscle to match experimentally measured room-temperature acoustic attenuation of rabbit muscle for this study. Tissue property databases show there can be property variations between species and even in different muscle groups or fiber orientations [66,67]. These differences are often smaller than the range of individual tissue measurements and within the uncertainty of the measurement techniques themselves. Errors introduced in FUS modeling from such uncertainties and differences could introduce biases into simulation-based treatment planning. This highlights the need for more comprehensive temperature-dependent property data in the literature as well as the potential benefit of noninvasive subject- or patient-specific property determination methods.

This study found that perfusion and acoustic attenuation are key properties in temperature-dependent FUS modeling. Characterization of perfusion, refinement in perfusion modeling and expanded experimental validation across tissue types and sonication parameters will enhance predictive accuracy of temperature-dependent models. Expanded efforts in characterizing temperature-dependent acoustic attenuation as well as acoustic absorption are also warranted.

## Conclusions

This study highlights the impact of integrating temperature-dependent properties in FUS simulations to improve the physical fidelity of treatment models. While peak temperature differences between constant and temperature-dependent models were small, cumulative effects on necrosis volumes were notable. Perfusion shutdown at high temperatures and hyperemia in moderate hyperthermia scenarios led to clinically relevant changes in the tissue thermal response. Thus, for both high-power ablative sonications and low-power hyperthermia scenarios, incorporating temperature-dependent tissue models is critical. Temperature-dependent property models may be less vital at moderate FUS powers. Because the temperature-dependencies of many tissues are unknown and may vary between individuals or within different regions, the results and conclusions of this study are based on the specific temperature-dependent models used. Other tissue and models may lead to different outcomes.

For FUS protocols involving continuous sonication with times of tens of seconds to minutes and powers of 3–60 acoustic watts, a 2.5-s update interval for temperature-dependent thermal property simulations provided a good tradeoff between simulation accuracy and computational efficiency. In the single case investigated, temperature-dependent acoustic attenuation greatly impacted temperature rise and necrosis volume predictions.

Generally, this study demonstrates that incorporating temperature-dependent properties need not require updates every time step for their effects to be accurately characterized. Incorporating these factors in simulations may aid simulation-based treatment planning efforts at optimizing target ablation, treatment duration, and healthy tissue preservation, supporting the continued development of FUS as a noninvasive therapy.

## Supplementary Material

Supp 1

Supplemental data for this article can be accessed online at https://doi.org/10.1080/02656736.2025.2606701.

## Figures and Tables

**Table 1.** Thermal and acoustic properties of liver, muscle, and other tissues used in FUS simulations.

Tissue type	Measurement Temperature (°C)	Specific Heat capacity (J/kg °C)	Thermal Conductivity (W/m °C)	Density (kg/m^3^)	Speed of Sound (m/s)	Acoustic Attenuation (Np/cm)	Perfusion (kg/ m^3^ s)

Liver	25	3620	0.54	1079	1586	0.069	15.05
Liver	37	3570	0.5	1079	1586	0.069	15.05
Muscle	20	3480	0.52	1090	1590	0.051	0.62
Muscle	37	3550	0.54	1090	1593	0.048	0.62
Water	–	4180	0.60	1000	1500	0.000	0.00
Fat	–	2350	0.54	911	1440	0.041	0.55
Bowel	–	3660	0.21	1088	1500	0.054	12.75
Bone	–	1330	0.32	1908	3515	0.513	0.17
Skin	–	3390	0.37	1109	1624	0.199	1.77

**Table 2.** Maximum temperature rise at sonication 5, necrosis volume, and computation time for each model in cases 1 (60 W) and 2 (30 W).

Case	Properties Model	Maximum Temperature Change (°C)	Necrosis Volume (mm^3^)	Computation Time (mm:ss)

Case 1	Constant at 25 °C	45.90	1208	17:27
	Constant at 37 °C	47.19	1275	19:08
	Temperature-dependent	47.02	1466	78:06
	Constant at 25 °C	22.55	96	17:00
Case 2	Constant at 37 °C	23.19	109	19:26
	Temperature-dependent	22.96	102	77:18

**Table 3.** Effect of thermal property update intervals on peak temperature, necrosis volume, and computation time in cases 1 (60 W) and 2 (30 W).

	Maximum Temperature Change (°C)		Necrosis Volume (mm^3^)		Computation Time (mm:ss)	
Update Interval (s)	Case 1	Case 2	Case 1	Case 2	Case 1	Case 2

0.1	47.02	22.96	1466	102	78:06	77:18
0.2	47.02	22.96	1466	102	51:33	51:02
0.5	47.02	22.95	1464	102	33:06	33:28
1.0	47.03	22.94	1462	102	28:40	29:38
2.5	47.04	22.92	1456	101	25:31	24:24
5.0	47.07	22.90	1444	100	24:53	24:47
10.0	47.08	22.86	1402	99	23:59	24:45
15.0	47.15	22.82	1348	99	24:14	24:48

**Table 4.** Effect of acoustic property update intervals on temperature rise and necrosis volume.

Update Interval (s)	Maximum Temperature Change (°C)	Necrosis Volume (mm^3^)	Computation Time (s)

0.05	26.16	21.375	82:53
0.10	26.15	21.375	47:02
0.25	26.12	21.250	21:52
0.50	26.08	21.000	15:43
1.25	25.94	20.625	08:43
2.50	25.71	20.125	08:32
5.00	25.21	19.000	08:14
7.50	24.79	17.000	07:49
10.00	24.34	15.500	07:44
Constant	18.23	3.125	06:22

## Data Availability

Data supporting the results and conclusions of this article will be made available by the authors upon request.
