# Supplementary material for: Integrating temperature-dependent tissue properties into focused ultrasound computational models for enhanced treatment planning
Source: Int J Hyperthermia. Author manuscript; Available in PMC 2026 Feb 2. (PMC12862921; doi:10.1080/02656736.2025.2606701)
Supplement: Supp 1 [file NIHMS2134585-supplement-Supp_1.docx]

**SUPPLEMENTAL MATERIAL**

Temperature-dependent property values as a function of the local tissue temperature are presented in Figure S1 through Figure S7.

**
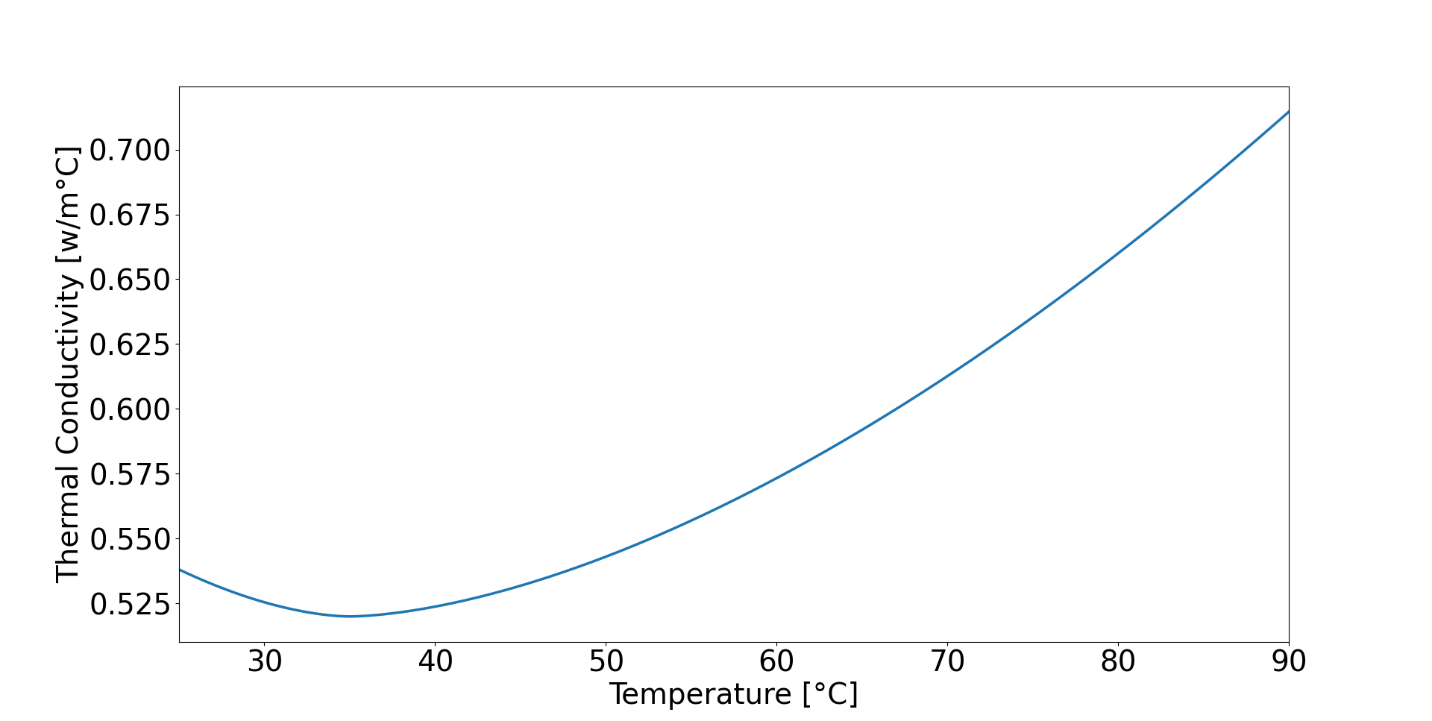
**

**Figure S1.** Temperature-dependent thermal conductivity of liver tissue.


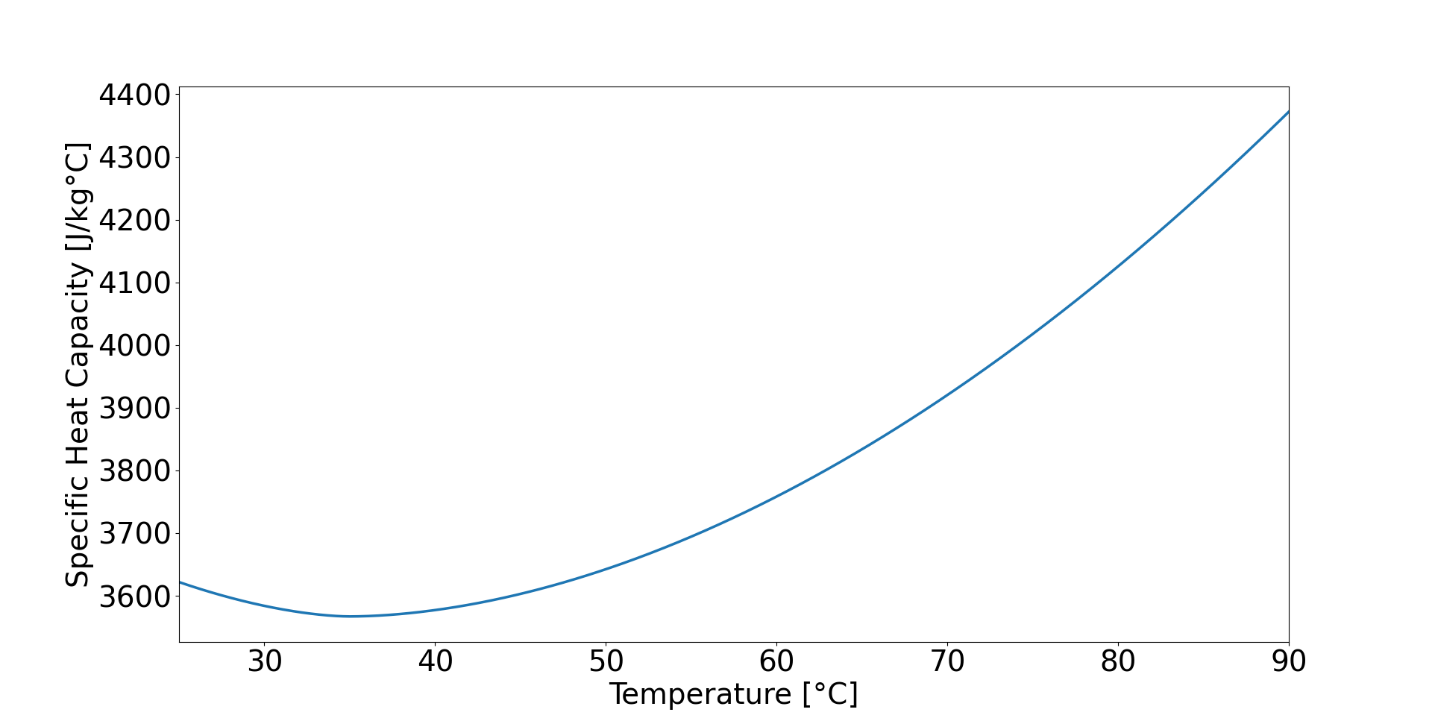


**Figure S2.** Temperature-dependent specific heat capacity of liver tissue.


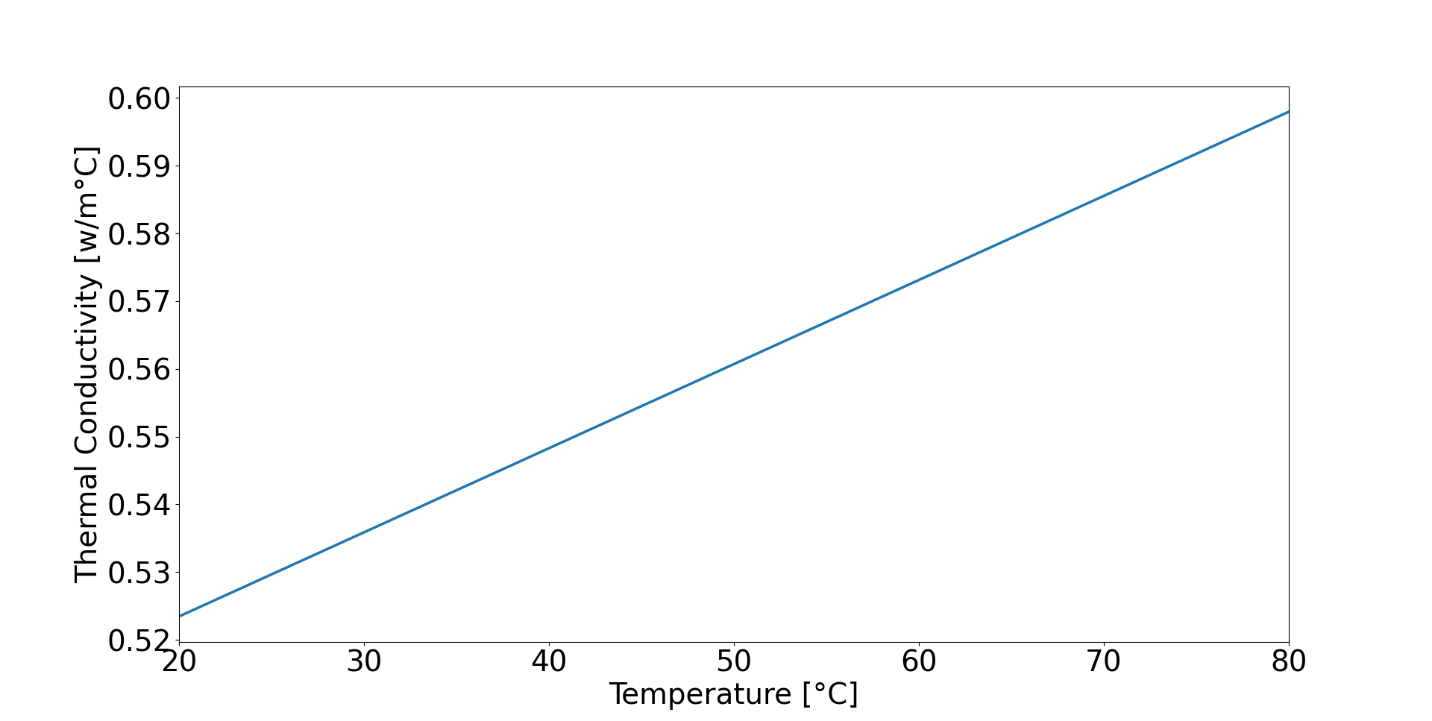


**Figure S3.** Temperature-dependent thermal conductivity of muscle tissue.


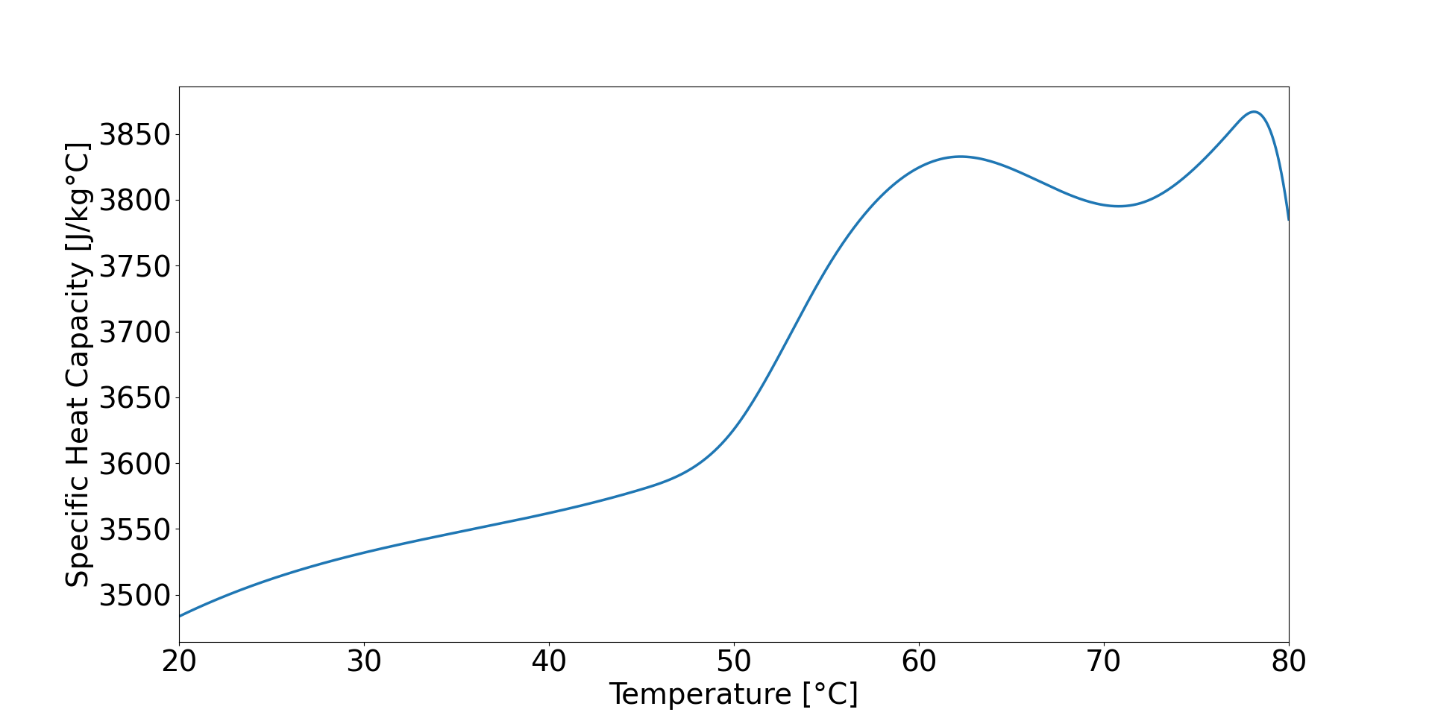


**Figure S4.** Temperature-dependent specific heat capacity of muscle tissue.


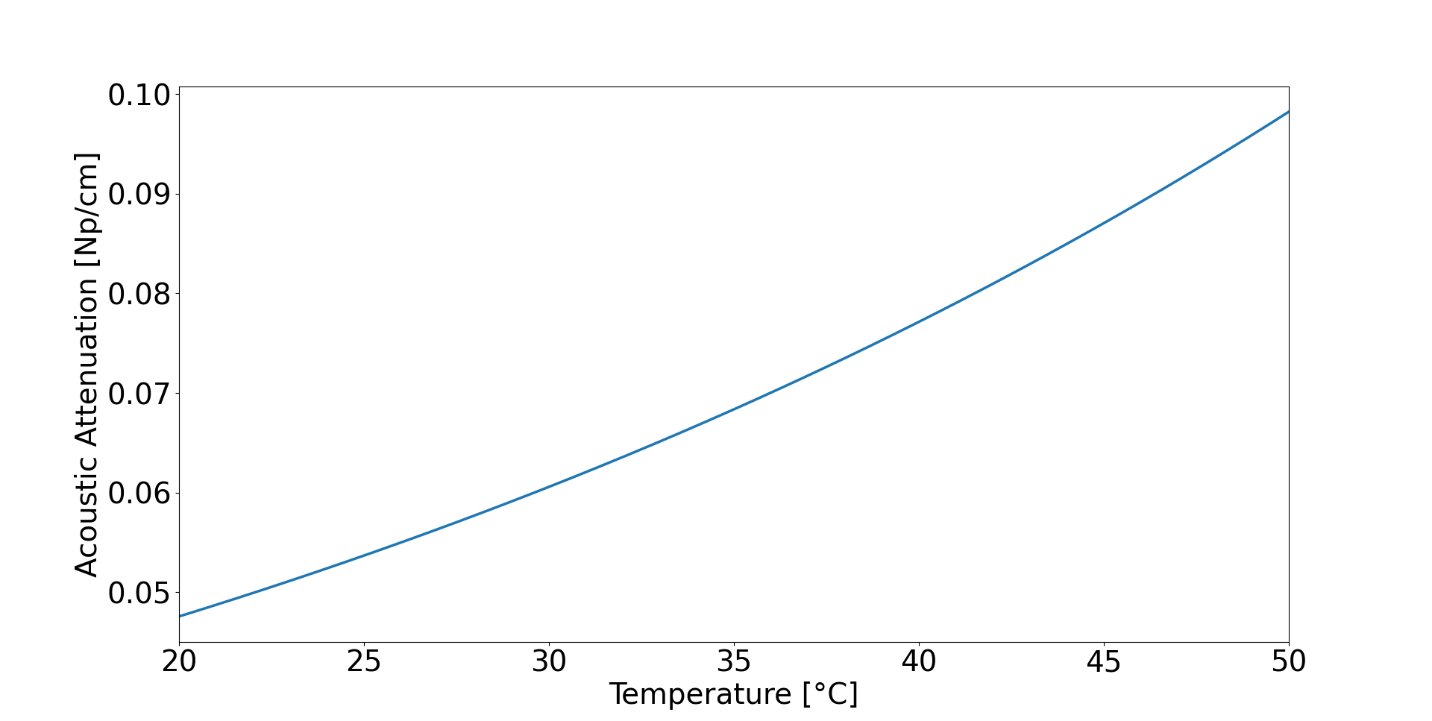


**Figure S5.** Temperature-dependent acoustic attenuation of muscle tissue.


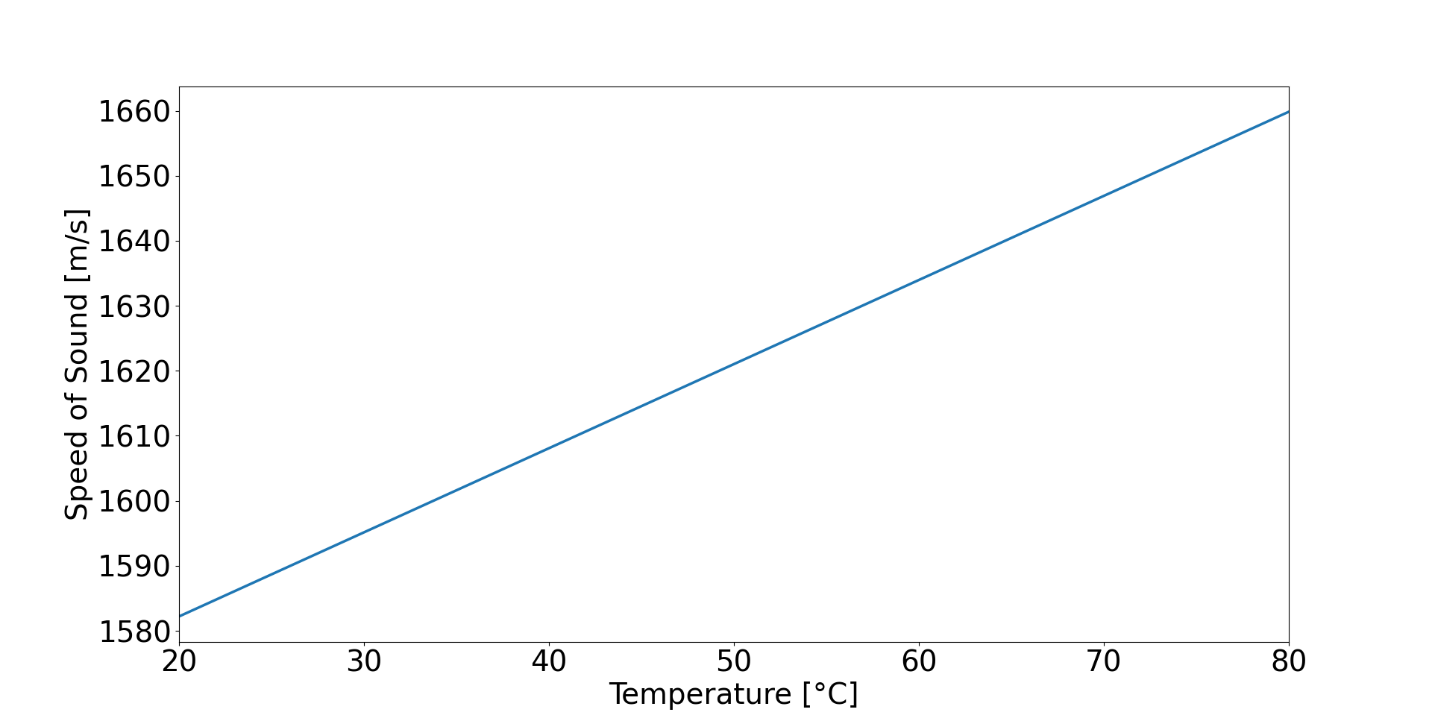


**Figure S6.** Temperature-dependent speed of sound of muscle tissue.


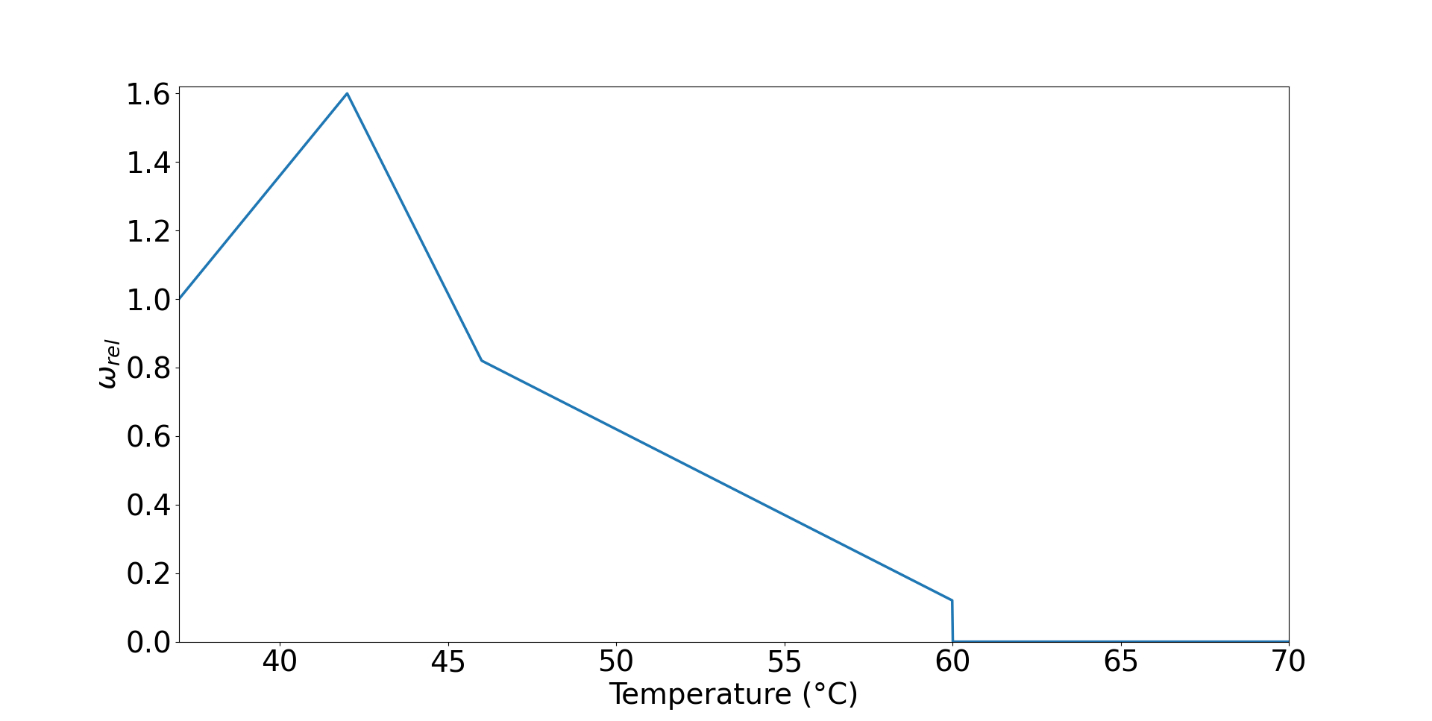


**Figure S7.** Relative perfusion factor ($\omega_{rel}$) values as a function of temperature.

Figure S8 highlights results from the liver tissue simulations with moderate powers (Scenario 1, Case 2). In these simulations, a transducer power of 30 W was applied for 30 seconds to each focal point with 30 seconds of cooling between sonications. The top plot of Figure S8 presents the temperature increase from 37 °C versus time at the center of Sonication 5 during its heating and cooling period (240-300 s) for the temperature-dependent model and the two constant-property models (37°C and 25°C). The inset graph in that top plot shows the temperature evolution for all nine focal locations, with the dashed line box indicating the period for the larger plot. With a more moderate power than Case 1, the temperature increase above 37 °C reached approximately 23 °C. The maximum variation between models was less than 0.64 °C for Case 2. The difference in cooling temperature profiles observed in Case 1 (see Figure 3) is not present for Case 2, because the temperature did not increase sufficiently to initiate perfusion shutdown in the simulations.

The middle row of Figure S8 displays the distribution of temperature increase from 37 °C in the transverse plane at 270 seconds (at the end of the heating period for sonication 5) for each property model. The bottom row shows the thermal dose map in the transverse plane for each property model. The total volume of necrotized tissue for Case 1 was 102 mm^3^ for the temperature-dependent tissue property model, with constant property models computing necrotized tissue volumes 6% smaller for properties at 25 °C and 7% larger for properties at 37 °C.


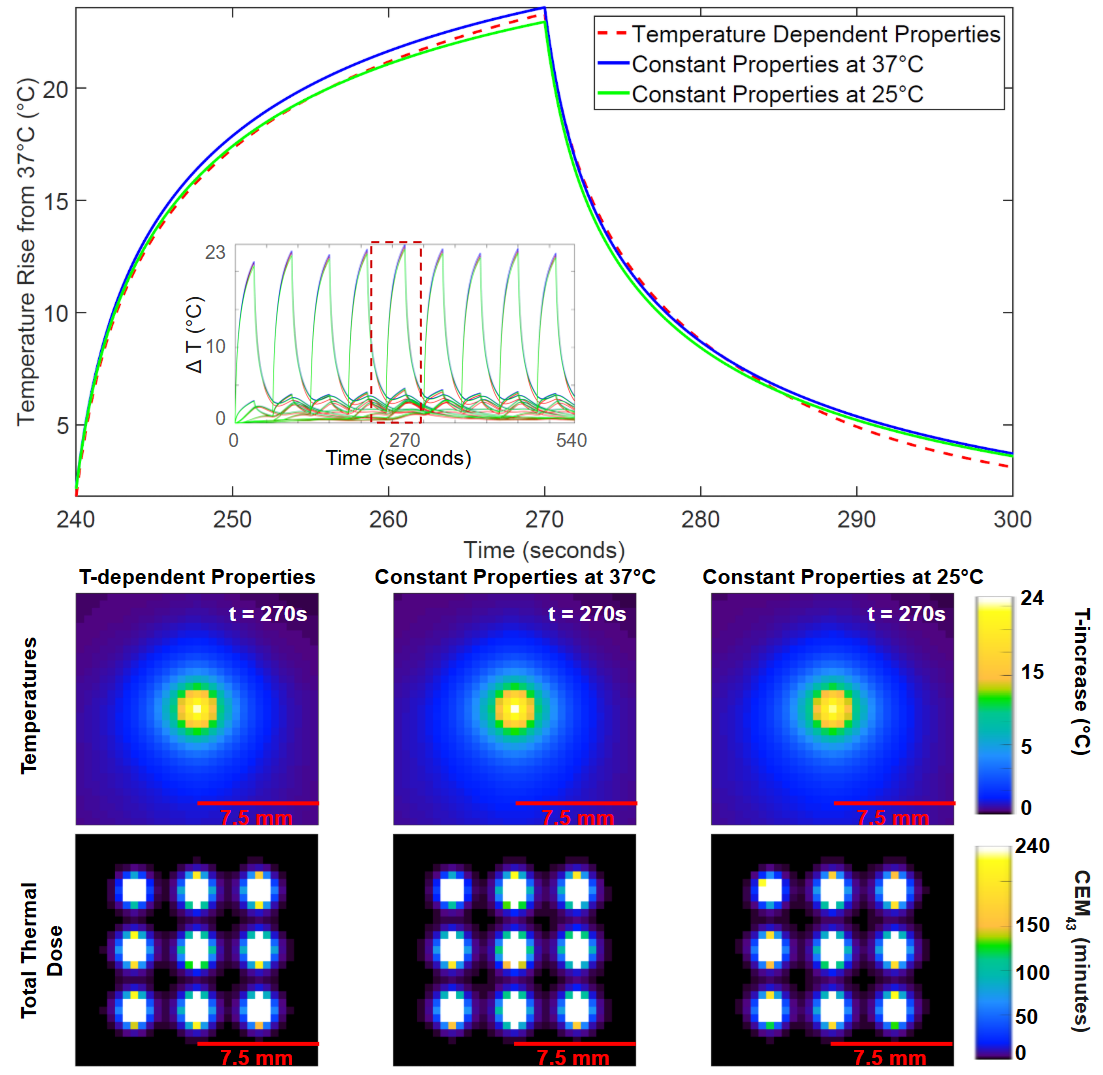


**Figure S8.** Temperature rise from 37 °C and thermal dose distribution for Scenario 1, Case 2 (30 W) in the liver tissue model.

Figure S9 distinguishes the contributions of temperature-dependent perfusion *ω* from those of specific heat capacity *c_p_* and thermal conductivity *k* on Scenario 1, Case 2 (30 W). Temperature-dependent *ω* with constant *c_p_* and *k* (dotted purple line) led to an increased temperature rise with a 110 mm^3^ total volume of necrotized tissue. Constant *ω* with temperature-dependent *c_p_* and *k* (dashed-dotted orange line) led to a decreased temperature rise with a necrotized tissue volume of 98 mm^3^. These results are like those of Scenario 1, Case 1 (60 W, Figure 4), except that the differences are less pronounced because the temperature increase and resulting property variations in Case 2 are smaller than in Case 1.


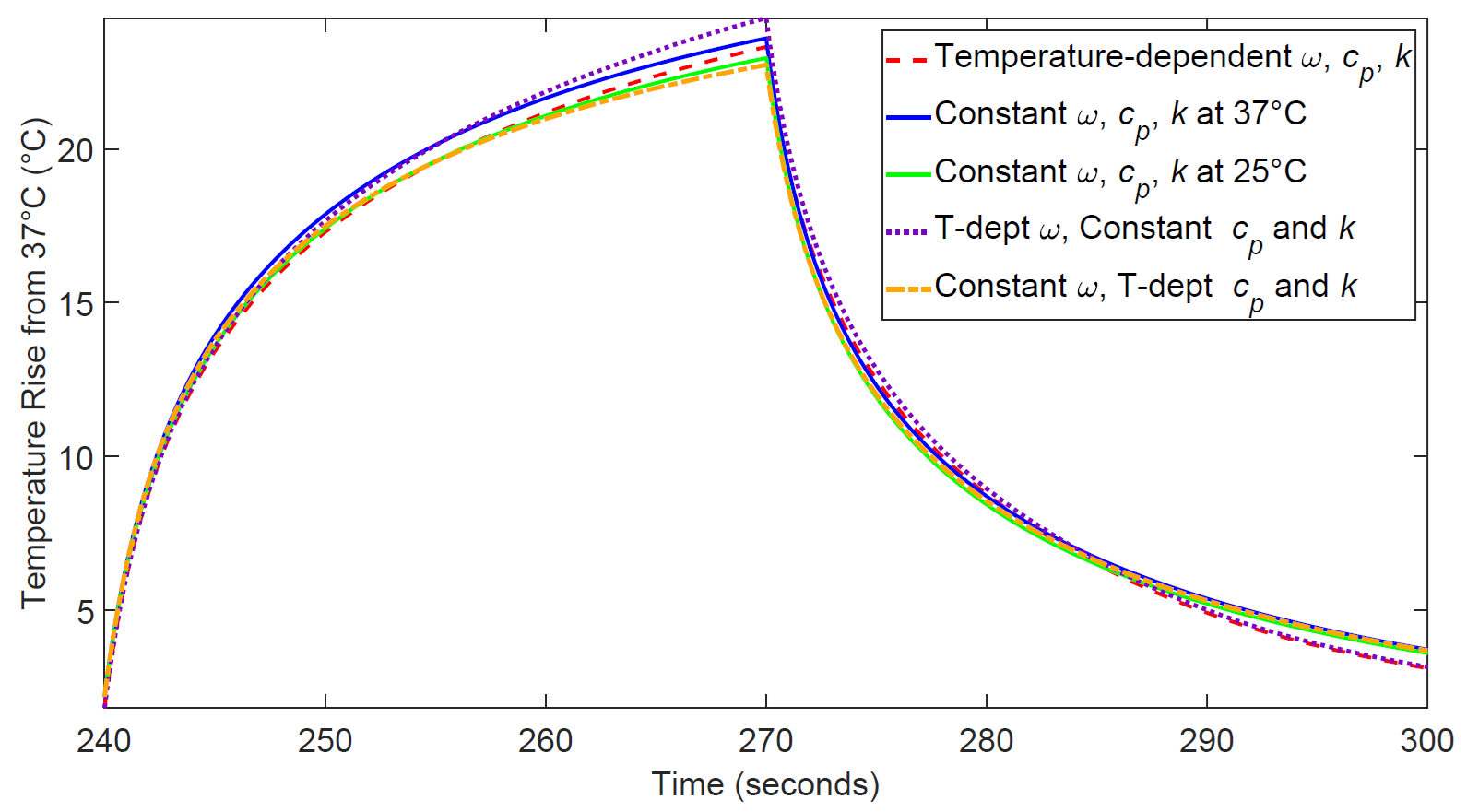


**Figure S9.** Temperature rise from 37 °C versus time for Scenario 1, Case 2 (30 W) utilizing various combinations of temperature-dependent and constant property models for perfusion *ω*, specific heat capacity *c_p_*, and thermal conductivity *k*.

Table S1 summarizes the time sensitivity analysis for Scenario 2, which modeled FUS heating of in vivo rabbit thigh muscle. The analysis focused on the first sonication of Rabbit 1, as it produced the highest temperature increase, offering the most significant opportunity to observe the effects of different update intervals. Minimal changes in both maximum temperature (<1% variation) and necrotized tissue volume (<3% variation) were observed across various update intervals.

**Table S1.** Effect of thermal property update intervals on temperature predictions, necrotized tissue volume, and computation time for Rabbit 1, Sonication 1.

| **Update Time (s)** | **Max Temperature Change (°C)** | **Necrotized Region (mm^3^)** | **Computation Time (mm:ss)** |
| --- | --- | --- | --- |
| 0.05 | 23.67 | 27.125 | 18:18 |
| 0.10 | 23.67 | 27.125 | 12:26 |
| 0.25 | 23.67 | 27.125 | 08:28 |
| 0.50 | 23.68 | 27.125 | 07:29 |
| 1.25 | 23.68 | 27.125 | 06:44 |
| 2.50 | 23.69 | 27.125 | 06:26 |
| 5.00 | 23.70 | 27.225 | 06:23 |
| 7.50 | 23.71 | 27.525 | 06:24 |
| 10.00 | 23.73 | 27.750 | 06:19 |

Similar to Scenario 1, the Scenario 2 time sensitivity analysis demonstrated that updating thermal properties at intervals up to 2.5 seconds is sufficient to maintain simulation accuracy for power levels up to ~50 W and temperatures up to 60°C. Updating properties every 2.5 seconds rather than every time step (0.05 seconds) enabled substantial reductions in computation time (63% reduction in computation time) without compromising accuracy.

Figure S10 focuses on Sonication 5 of Rabbit 2, displaying the temperature distribution at the end of heating, and concurrent property distributions for specific heat capacity *c_p_*, perfusion *ω*, and thermal conductivity *k*. Because the temperature rise for this low-power sonication was only 6 °C, the changes in *c_p_* and *k* were small with deviations from baseline values of just a few percent. Perfusion *ω* on the other hand, exhibited a relatively large increase near the focal region where hyperthermic temperature increase blood flow according to Eq. 3.


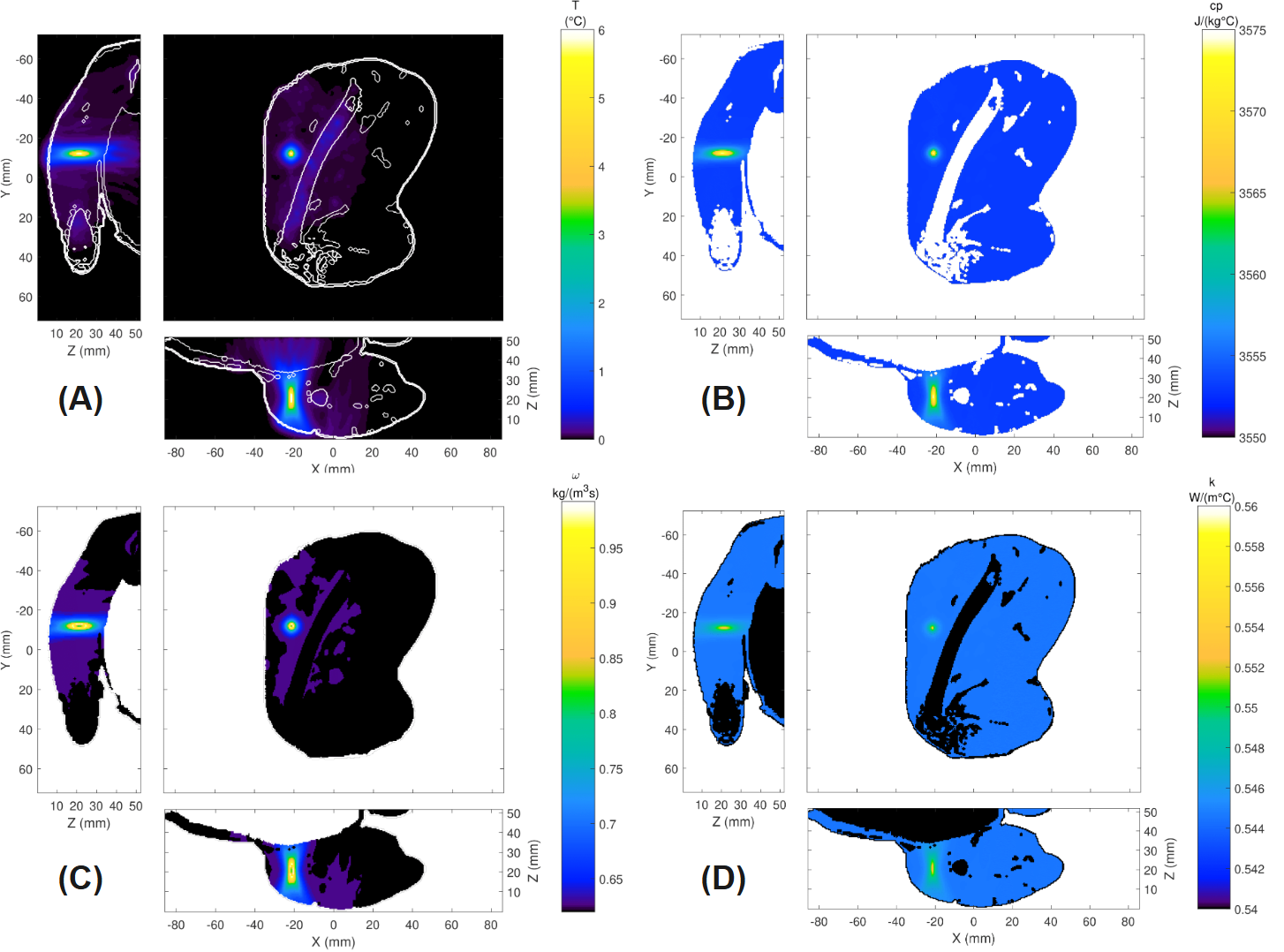


**Figure S10.** Spatial distribution of temperature rise from 37 °C and thermal properties at the end of heating for Rabbit 2, Sonication 5. (A) Temperature increase, (B) Specific heat capacity *c_p_*, (C) Perfusion *ω*, and (D) Thermal conductivity *k*.

Table S2 summarizes results from each of the nine sonications in rabbit thigh muscle for Scenario 2 with each of the three property models. These results used an update interval of 1.25 s, which resulted in ~17% longer computation times for the temperature-dependent property model compared to constant property models.

**Table S2.** Comparison of maximum temperature change, necrotized tissue volume, and computation time for each of the three property models and all nine sonications of Scenario 2 in rabbit thigh muscle.

| **Rabbit, Location** | **Model** | **Maximum Temperature Change (°C)** | **Necrotized Region (mm^3^)** | **Computation Time (m:ss)** |
| --- | --- | --- | --- | --- |
|  | Constant properties at 20°C | 25.1 | 32 | 5:40 |
| **1, 1** | Constant properties at 37°C | 24.2 | 28 | 5:45 |
|  | Temperature-dependent properties | 23.7 | 27 | 6:44 |
|  | Constant properties at 20°C | 21.4 | 11 | 5:54 |
| **1, 2** | Constant properties at 37°C | 20.7 | 9 | 5:43 |
|  | Temperature-dependent properties | 20.2 | 8 | 6:39 |
|  | Constant properties at 20°C | 21.5 | 10 | 5:40 |
| **1, 3** | Constant properties at 37°C | 21.0 | 9 | 5:49 |
|  | Temperature-dependent properties | 20.4 | 7 | 6:48 |
|  | Constant properties at 20°C | 21.1 | 14 | 5:44 |
| **1, 4** | Constant properties at 37°C | 20.4 | 11 | 5:45 |
|  | Temperature-dependent properties | 20.0 | 10 | 6:41 |
|  | Constant properties at 20°C | 4.0 | 0 | 5:37 |
| **2, 1** | Constant properties at 37°C | 3.8 | 0 | 5:46 |
|  | Temperature-dependent properties | 3.8 | 0 | 6:37 |
|  | Constant properties at 20°C | 6.5 | 0 | 5:44 |
| **2, 2** | Constant properties at 37°C | 6.3 | 0 | 5:48 |
|  | Temperature-dependent properties | 6.3 | 0 | 6:42 |
|  | Constant properties at 20°C | 6.3 | 0 | 5:39 |
| **2, 3** | Constant properties at 37°C | 6.1 | 0 | 5:51 |
|  | Temperature-dependent properties | 6.1 | 0 | 6:50 |
|  | Constant properties at 20°C | 6.3 | 0 | 5:43 |
| **2, 4** | Constant properties at 37°C | 6.1 | 0 | 5:49 |
|  | Temperature-dependent properties | 6.1 | 0 | 6:45 |
|  | Constant properties at 20°C | 6.8 | 0 | 5:38 |
| **2, 5** | Constant properties at 37°C | 6.6 | 0 | 5:47 |
|  | Temperature-dependent properties | 6.5 | 0 | 6:39 |
